# The multifaceted role of extracellular vesicles (EVs) in colorectal cancer: metastasis, immune suppression, therapy resistance, and autophagy crosstalk

**DOI:** 10.1186/s12967-024-05267-8

**Published:** 2024-05-13

**Authors:** Soheil Rahmati, Aysan Moeinafshar, Nima Rezaei

**Affiliations:** 1 Student Research Committee, Ramsar Campus, Mazandaran University of Medical Sciences, Ramsar, Iran; 2 Universal Scientific Education and Research Network (USERN), Tehran, Iran; 3 School of Medicine, Tehran University of Medical Sciences, Tehran, Iran; 4 Research Center for Immunodeficiencies, Children’s Medical Center, Tehran University of Medical Sciences, Dr. Qarib St, Keshavarz Blvd, Tehran, 14194 Iran; 5 Network of Immunity in Infection, Malignancy, and Autoimmunity (NIIMA), Universal Scientific Education and Research Network (USERN), Tehran, Iran; 6 Department of Immunology, School of Medicine, Tehran University of Medical Sciences, Tehran, Iran

**Keywords:** Extracellular vesicles, Exosomes, Colorectal cancer, Metastasis, Immune suppression, Therapy resistance, Autophagy, miRNAs, Biomarkers

## Abstract

Extracellular vesicles (EVs) are lipid bilayer structures released by all cells and widely distributed in all biological fluids. EVs are implicated in diverse physiopathological processes by orchestrating cell–cell communication. Colorectal cancer (CRC) is one of the most common cancers worldwide, with metastasis being the leading cause of mortality in CRC patients. EVs contribute significantly to the advancement and spread of CRC by transferring their cargo, which includes lipids, proteins, RNAs, and DNAs, to neighboring or distant cells. Besides, they can serve as non-invasive diagnostic and prognostic biomarkers for early detection of CRC or be harnessed as effective carriers for delivering therapeutic agents. Autophagy is an essential cellular process that serves to remove damaged proteins and organelles by lysosomal degradation to maintain cellular homeostasis. Autophagy and EV release are coordinately activated in tumor cells and share common factors and regulatory mechanisms. Although the significance of autophagy and EVs in cancer is well established, the exact mechanism of their interplay in tumor development is obscure. This review focuses on examining the specific functions of EVs in various aspects of CRC, including progression, metastasis, immune regulation, and therapy resistance. Further, we overview emerging discoveries relevant to autophagy and EVs crosstalk in CRC.

## Introduction

Colorectal cancer (CRC) is the third most commonly diagnosed cancer and the second leading cause of cancer deaths worldwide [1]. CRC is more frequently shifting to diagnosis at a younger age and in a more advanced stage [2]. Metastasis and recurrence are the main causes of death in CRC. In localized disease, the 5-year survival rate is 91%, with a decrease to 14% in remote metastases [3]. Despite advances in CRC screening, a subgroup of patients is initially diagnosed with metastatic disease, which highlights the requirement for new diagnostic and prognostic methods [2].

EVs are heterogenous lipid bilayers, dischargeable by any cell, present in all biological fluids, and lack the ability to reproduce. Based on their cellular origin, they differ in size, mechanism of formation, membrane composition, and content. Therefore, they are mainly categorized into three groups: exosomes, microvesicles, and apoptotic bodies [4–6]. Exosomes have an endosomal origin and range from 40 to 160 nm in size, formed by double budding of the plasma membrane, followed by their excretion into the extracellular space. EVs are known to be involved in intercellular communications and have been implicated in different diseases, particularly cancer [7].

Colorectal cancer cells interact with themselves and stromal cells by releasing EVs in the tumor microenvironment. Literature has suggested that CRC-derived EVs can alter the tumor microenvironment (TME) to cause tumor progression and metastasis [8, 9]. EVs mainly function by transferring their cargo, which encompasses nucleic acids, proteins, and lipids, to target cells. As a result, they influence and regulate the physiological function of the recipient cells [9]. For instance, it has been shown that EVs play an essential role in inducing epithelial-mesenchymal transition (EMT) in CRC cells, initiating extracellular matrix degradation, premetastatic niche formation, and angiogenesis to facilitate metastasis [10]. In addition, CRC-derived EVs are involved in immune evasion and tumor resistance by recruiting suppressive immune cells (Tumor-associated macrophages, cancer-associated fibroblasts, regulatory T-cells, and myeloid-derived suppressor cells) in TME [8]. A growing body of studies has been focused on the potential of EV cargoes as diagnostic and prognostic biomarkers in malignancies, including CRC. Moreover, the potential of EVs to transport therapeutic drugs to target cancer cells positions them as promising vehicles for drug delivery [6, 7]. Autophagy is a conserved recycling process that catabolizes damaged intracellular components and recycling them via the lysosomal-dependent pathway [11]. The EV release pathway, combined with the process of autophagy, works in tandem to maintain cellular homeostasis and protect cellular integrity during stressful conditions. Vesicular trafficking is implicated in both mechanisms, and autophagy plays a critical role in the generation and breakdown of EVs. The relationship between autophagy machinery and EV release pathway has recently attracted much attention since their coordination may have profound implications for human diseases, including cancer [12, 13].

In the current review, the effects of EVs on CRC metastasis, immune regulation, therapy resistance, and their clinical application in CRC diagnosis and treatment, and finally, emerging studies on autophagy and EVs crosstalk will be discussed.

## EVs biogenesis, cargo, and secretion

All cells, including almost all forms of life from prokaryotes to eukaryotes, can release different types of membrane vesicles known as extracellular vesicles (EVs) as part of their normal physiological function and during pathological state. EVs were initially considered cellular waste to eliminate unwanted materials. Nonetheless, now EVs are recognized as participants in myriad cellular processes and hold a pivotal position in intercellular communication owing to their ability to transport their contents, such as lipids, proteins, RNAs, and DNA, to neighboring or remote cells [5]. In 1983, two groups independently discovered that transferring receptors associated with small membrane vesicles are released by maturing reticulocytes into the extracellular space. Johnstone called these nano-sized vesicles exosomes [14–16]. The term “extracellular vesicles” currently refers to all lipid bilayer structures secreted by cells and widely distributed in all biological fluids [4, 5]. EVs are distinct in size, cell of origin, biogenesis mechanism, and content, causing their diversity. EVs characterization is continuously evolving. Based on the International Society of Extracellular Vesicles, EVs can be classified into two categories: small EVs (sEVs) measuring less than 200 nm in diameter and large EVs with a diameter exceeding 200 nm [4]. Alternatively, they can be further categorized as exosomes, microvesicles, and apoptotic bodies [6]. Recently discovered nanoparticles, known as exomeres and supermeres, possess functional properties but lack membranous bilayers [17, 18].

Exosomes, which have a diameter ranging from 40 to 160 nm, are formed through the double invagination of the plasma membrane. This process leads to the generation of intraluminal vesicles (ILVs) within the multivesicular body (MVB). First invagination forms the early endosome containing cell surface proteins and extracellular materials, and second budding leads to generation of ILVs within the late endosome. During this process, cytosolic proteins and nucleic acids can become enclosed in ILV, which is facilitated by the endosomal sorting complex required for transport (ESCRT) [7]. ESCRT is composed of four complexes, ESCRT-0, -I, -II, and -III, which regulate ILV formation and sort cargoes into specific microdomains of the MVB’s limiting membrane [5]. The ESCRT machinery operates in a sequential manner. Phosphatidylinositol 3-phosphate activates ESCRT-0, which then recruits ESCRT-I to gather ubiquitinated transmembrane proteins on MVB microdomains. ESCRT-I recruits ESCRT-II, and both drive inward budding of the late endosome membrane, triggering ESCRT-III activation to cleave the endosomal membrane [5]. The final step requires ESCRT-III interaction with the AAA ATPase Vps4 for de-ubiquitination of cargoes and ESCRT-III detachment from the endosomal membrane [19, 20].

MVB can undergo one of two outcomes: either it can merge with the plasma membrane (PM) and discharge exosomes into the extracellular space, or merge with lysosomes and break down its contents [5, 7]. Rab-GTPase families, including Rab7, 11, 35, and 27A /B, participate in MVB trafficking toward the plasma membrane or lysosome. Rab7 transports MVB toward the lysosome, Rab11 and Rab35 regulate endosomal membrane components’ recycling, and Rab27A and Rab27B promote MVB docking to PM [5, 21, 22]. SNARE proteins such as VAMP7 and YKT6 are implicated in driving MVB fusion with PM [23, 24]. Hessvik et al. have reported that depletion of SNAP29, VAMP8, STX18, STX2, and STX3 reduced exosome secretion in the PC-3 prostate cancer cell line. Among these SNARE proteins, SNAP29 depletion reduced exosome release in three other cancer cell lines, suggesting that SNAP29 plays a general role in exosome secretion. However, depletion of SNAP29 did not affect ectosome release and secretory autophagy [25]. It has been evidenced that MVB formation and exosome release also occur in an ESCRT-independent pathway, which is mediated through syndecan-syntenin-ALIX pathway, ceramide, tetraspanin families (CD 63, 81), heat shock proteins (HSP70), and tumor susceptibility 101 (TSG 101) [5, 21].

Following their release from donor cells into the extracellular space, exosomes have two potential pathways: either they can be internalized by neighboring cells or navigate through the circulatory system to reach remote recipients. Depending on the origin and identity of exosomes and their target cells, exosomes can affect the function of recipient cells by direct ligand-receptor interaction, fusion with the plasma membrane, or endocytosis. However, the principles of exosome uptake and their intercellular trafficking are yet to be elucidated [5].

**Fig. 1:**
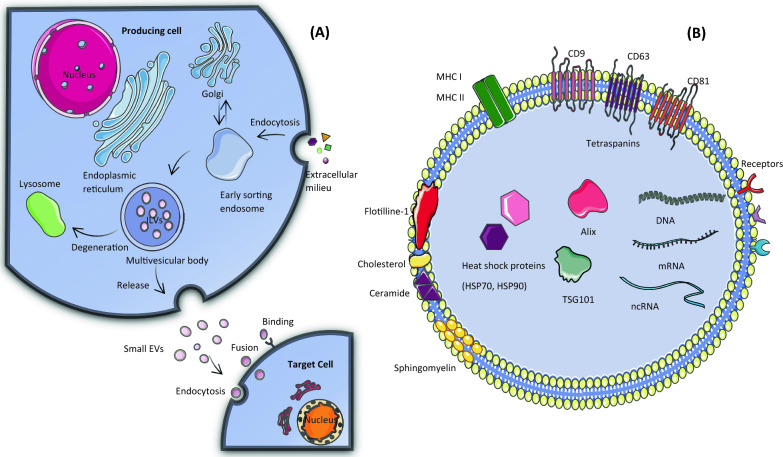
**A** sEVs biogenesis, release, and interaction with the target cell. EVs are created through a double inward budding of the plasma membrane. The initial budding results in the generation of an early sorting endosome, which embeds extracellular milieu and cell surface proteins. Subsequently, a second budding occurs within the late endosome, leading to the generation of intraluminal vesicles (ILVs). The multivesicular body (MVB) has two pathways it can follow: it can merge with the plasma membrane, leading to the release of extracellular vesicles (EVs) into the surrounding space, or it can combine with the lysosome to degrade its contents. Upon release into the extracellular space, EVs possess the capacity to impact the functionality of their target cells through direct ligand-receptor binding, fusion, or endocytosis. **B** EV cargoes and components. EVs facilitate the transfer of diverse molecules, including DNAs, mRNAs, ncRNAs, proteins, and lipids [5, 7]. The figure was designed using graphical elements from Servier Medical Art, made available by Servier under a Creative Commons Attribution 3.0 unported license. (https://creativecommons.org/licenses/by/4.0/)

Microvesicles, also called ectosomes, have a diameter that varies from 50 nm to 1 μm and are shed by direct outward budding of the plasma membrane [5]. Apoptotic bodies are bigger vesicular structures with a size of 800 nm to 5 μm that form in a similar fashion during the apoptotic process, carrying cellular organelles and DNA fragments [6]. Exomeres are 35 nm diameter non-membranous newly discovered extracellular particles that can be separated via asymmetric flow field-flow fractionation (AF4) and sequential high-speed ultracentrifugation. They possess a significant abundance of metabolic enzymes participating in glycolysis, as well as lipids, and nucleic acids [17]. Supermeres are supernatants of exomeres with distinct proteins and RNA profiles and significantly higher uptake than exomeres and exosomes in vivo. Most extracellular RNAs are associated with supermeres compared to other extracellular particles. Among these RNAs, miR-1246 is most differentially expressed in supermeres derived from CRC cell line, DiFi [18] (Fig. 1).

## Role of sEVs in CRC progression and metastasis

Metastasis accounts for the majority of mortality in CRC patients, with the liver being the most common site of metastasis [26, 27]. Population-based studies have demonstrated approximately 25–30% of CRC patients will develop liver metastasis over the course of their life [28]. These data point to the necessity of understanding the underlying principles of metastasis.

**Fig. 2:**
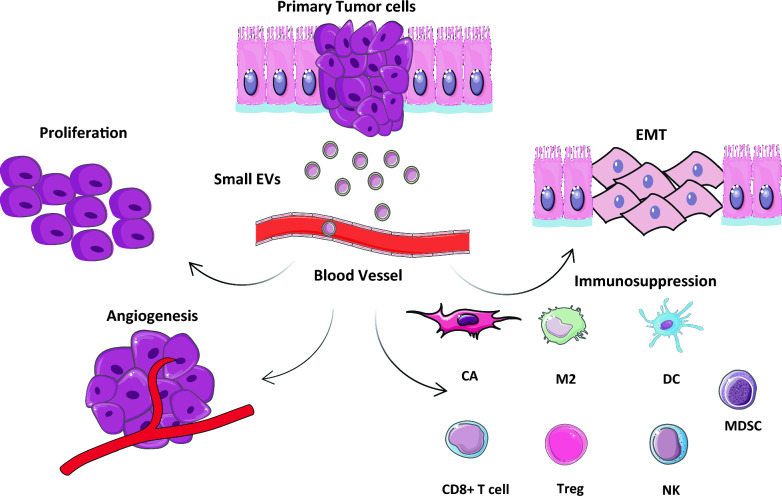
The metastasis of colorectal cancer (CRC) is facilitated by tumor-derived small extracellular vesicles (sEVs). These sEVs travel through the bloodstream and coordinate the spread of cancer to secondary sites through various mechanisms, including epithelial-mesenchymal transition (EMT), creating a premetastatic environment, promoting immunosuppression, and stimulating angiogenesis and the proliferation of CRC cells. The figure was designed using graphical elements from Servier Medical Art, made available by Servier under a Creative Commons Attribution 4.0 unported license. (https://creativecommons.org/licenses/by/4.0/)

CRC cells are in continuous interplay with their surrounding cells in TME, which is orchestrated by EVs [9]. Accumulative studies have demonstrated CRC-derived EVs can alter TME to favor tumor progression, premetastatic niche formation, metastasis, and angiogenesis (Fig. 2).

### sEVs effects on epithelial-mesenchymal transition (EMT)

Metastasis consists of a series of sequential steps involving tumor cell invasion through tumor stroma, dissemination in circulation, extravasation, and colonization at the secondary site [29]. EVs have a critical role in the process of metastasis by driving EMT and extracellular matrix (ECM) degradation [30]. EMT refers to a process in which tumor cells undergo a transformation, relinquishing their epithelial features and adopting mesenchymal properties [31]. This is accompanied by conversion to a low proliferation state and turmoil of apical-basal polarity and cell–cell junctions, promoting motility and invasion of tumor cells. In turn, the reverse phenomenon, referred to as Mesenchymal-epithelial transition (MET), takes place once tumor cells reach a distant premetastatic site, enabling the formation of micrometastases [32]. During EMT, there is documented evidence of reduced expression of epithelial markers, such as E-cadherin, β-catenin, and claudins. Subsequently, there is an observed elevation in the expression of mesenchymal markers, such as vimentin, N-cadherin, and fibronectin [33]. Abundant signaling pathways (including Wnt, TGF-β, p53, mTOR, MAPK, and JAK-STAT) and multiple transcriptional factors (such as TWIST, SNAIL, Slug, FOX, and zinc-finger E-box binding (ZEB)) have been described to modulate EMT. EVs derived from diverse cells in CRC microenvironment have been implicated in initiating metastasis and driving EMT [34–36].

#### The effect of tumor cell-derived EVs on EMT

Frizzled (FZD) proteins are a group of membrane receptors that are integral components of the WNT signaling pathway. Scavo and colleagues portrayed exosomes derived from CRC cells harboring FZD10 trigger EMT in normal colon epithelial cells [37]. Furthermore, Scavo’s team, in another study, reported FZD10-mRNA silenced cells showed reduced viability compared to the respective controls. Notably, when the silenced cells were exposed to exosomes isolated from untreated cell lines that contained FZD10 and FZD10-mRNA, it led to an improved recovery of cell viability [38]. In addition, Liang and coauthors reported lncRNA RPPH1 is highly expressed in SW620 and HCT8 cells and escalates malignant cell migration ability and EMT by physically binding to β-III tubulin (TUBB3) [39].

Yang et al. revealed exosomal transfer of microRNA-106b-5p (miR-106b) originating from CRC cells which have undergone the EMT process, induce M2-like polarization by suppressing programmed cell death 4 (PDCD4) and activating PI3Kγ/Akt/mTOR axis. Reciprocally, M2 macrophages enhance the migration of CRC cells by promoting the process of EMT [10]. Aberrant CXCR7 expression in CRC cells promotes CAFs activation via transferring miR-146a-5p and miR-155-5p to fibroblast, triggering EMT and metastasis. Mechanistically, miR-155-5p and miR-146a-5p negatively regulate the suppressor of cytokine signaling 1 (SOCS1) and the zinc finger and BTB domain containing 2 (ZBTB2), respectively. Targeting SOCS1 and ZBTB2 leads to subsequent activation of CAFs through JAK–STAT3/NF-κB signaling. Moreover, in tumor xenograft models, transfected CAFs with these miRNAs facilitate lung metastasis [40].

#### The role of non-tumor cell-derived EVs on EMT

Aside from CRC cells, EVs derived from stromal and immune cells contribute to metastasis [41]. EVs with high levels of miR-186-5p originating from M2 macrophage expedite colon cancer cell expansion and motility through downregulating DLC1 expression [42]. Lan et al. reported that exosomes isolated from M2 macrophage harbor miR-21-5p and miR-155-5p, which trigger CRC migration and invasion via downregulating the tumor suppressor BRG1 expression [43]. The Wnt/β-catenin pathway is acknowledged as a fundamental mechanism that plays a pivotal role in promoting EMT [44]. Macrophage-derived EVs containing Wnt can induce β-catenin, leading to intestinal stem cell maintenance and epithelial repair [35].

Cancer-associated fibroblasts (CAFs), vital constituents of TME, have been shown to play dominant roles in tumor metastasis through EMT regulation and ECM reorganization. By stimulating the Wnt/β-catenin signaling and hampering mitochondrial apoptosis, exosomal miR-92a-3p derived from CAFs directly promotes CRC cell stemness, metastasis, and resistance to 5-Fluorouracil (5-FU)/oxaliplatin. Indeed, exosomal miR-92a-3p exerts this oncogenic role by suppressing its downstream targets F-box and WD repeat domain containing 7 (FBXW7) and modulator of apoptosis 1 (MOAP1) [34]. LINC00659, originating from CAFs, is delivered to tumor cells and functions as a miR-342-3p sponge, thereby escalating annexin A2 (ANXA2) expression in CRC cells. This contributes to proliferation of tumor cells, invasion, and progression of EMT [45].

### sEVs effects on PMN formation

Primary tumor creates a conducive microenvironment known as premetastatic niche (PMN) in the secondary site, facilitating the settlement and growth of disseminated malignant cells. The selection of a specific organ for remote metastasis is not a random occurrence but rather a result that is partially modified by the primary tumor [46]. Primary tumor secretory factors, including EVs, prepare the distant site for subsequent metastasis [47]. Unique features of the premetastatic niche (PMN) have been described as crucial for creating an effective environment where cancer cells can settle and proliferate. These features encompass angiogenesis and vascular permeability, inflammation, immunosuppression, lymphangiogenesis, reprogramming, and organotropism. Interaction between primary tumor-derived factors, recruited bone marrow-derived cells (BMDCs), and local stromal microenvironment leads to PMN formation [47].

miR-25-3p, originating from CRC cells, facilitates the establishment of PMN via induction of vascular leakiness and angiogenesis by silencing transcription factors Krüppel-like factor 2 and 4. Krüppel-like factor 2 (KLF2) attenuates angiogenesis by inhibiting the promoter activity of VEGFR2. On the other hand, KLF4 takes part in upholding endothelial barrier stabilization by enhancing the expression of tight junction proteins, including ZO-1, occludin, and Caludin5. In addition, miR-25-3p derived from CRC cells was associated with liver and lung metastasis in nude mice [48]. Exosomal transfer of interferon regulatory factor 2 (IRF2) augments VEGFC secretion by sentinel lymph node (SLN) macrophage, leading to lymphangiogenesis and SLN metastasis of colon cancer [49].

Modification of ECM amplifies the adherence of circulating tumor cells (CTCs), such as through the facilitation of heightened accumulation of fibronectin in the liver. Pancreatic ductal adenocarcinoma (PDAC)-derived exosomes enriched in macrophage migration inhibitory factor (MIF) promote activation of hepatic stellate cells (HSCs) and ECM remodeling via inducing TGF- β secretion in Kupffer cells. Activated HSCs lead to fibronectin accumulation and enhanced recruitment of bone marrow-derived macrophages, together contribute to PMN formation in the liver [50]. Similarly, EV-associated transfer of miR-181a-5p from CRC cells with high metastatic capability facilitates TME remodeling, PMN formation, and liver metastasis. This occurs by stimulation of HSCs by dampening the expression of SOCS3 and boosting IL6/STAT3 pathway [51]. Moreover, EV-miR-320c expression in mCRC cells mediates mesenchymal-epithelial transition programming and creates tumor-favoring metastatic niche [52].

Primary cancer cell-derived factors and EVs stimulate the mobilization of BMDCs and other immune cells with suppressive properties, such as tumor-associated macrophages (TAMs), regulatory T cells (Tregs), and tumor-associated neutrophils (TANs), into the PMN to form an immunosuppressive microenvironment [47]. Exosomal transfer of mir-934 prompts differentiation of macrophages into a pro-tumorigenic phenotype by suppressing PTEN expression and stimulating the PI3K/AKT signaling. In turn, M2 macrophages facilitate liver metastasis by augmenting CXCL13/CXCR5 axis in CRC cells [53]. Hypoxia-induced exosomal miR-135a-5p orchestrates a hepatic PMN by simultaneously modulating suppression of the immune system and cell adhesion. Mechanistically, miR-135a-5p exerts its immunosuppressive effect by impairing CD30-mediated CD4 + T cell activation and improving cell adhesion through an increase in MMP7 expression [54]. Yang and colleagues unveiled the mechanism of CRC-derived EVs in conducting immunosuppressive PMN in early liver metastasis. Interestingly, EVs harboring transforming growth factor-β (TGF-β1) provoke HSCs differentiation into CAFs by activating HSCs chemokines signaling. Moreover, myeloid-derived suppressor cells (MDSCs) are further recruited to abrogate NK cell cytotoxicity by repressing NKG2D expression [55].

While chronic inflammation drives colon tumorigenesis to a large extent, inflammation can either promote or inhibit the growth and viability of malignant cells [56]. The regulatory effects of inflammatory cells on initiation and progression of CRC chiefly hinge on the composition of immune infiltrate and the synthesis of distinct cytokines and chemokines [57]. Cytotoxic T lymphocytes and natural Killer (NK) cells display anti-tumor response, while M2 macrophages, Tregs, and CD4 + T helper (Th)-17 cells exert tumor-promoting activities. Furthermore, cytokines such as IL-6, IL-21, IL-17A, and tumor necrosis factor-α (TNF-α) induce tumor-supportive microenvironment, while interferon-γ (IFN-γ) exerts tumor-suppressive response [57, 58].

IL-9 has been regarded as a pro-tumorigenic and anti-tumorigenic cytokine in hematological malignancies and solid tumors, respectively [59]. Recent discoveries have demonstrated both the pro-tumorigenic and anti-tumorigenic functions of IL-9 in CRC [60–62]. Gerlach et al. presented that mucosal Th9 cells producing IL-9 are abundant in CRC specimens and accelerate the proliferation of intestinal epithelial cells through positive regulation of IL-6 production derived from T cells [60]. Another study indicated low expression of IL-9 in CRC tissues and suggested an anti-tumorigenic effect of IL-9 through the facilitation of CD8 + T cell infiltration and the creation of an anti-tumor microenvironment [61].

By activating fibroblasts in remote organs, EVs-transferred integrin beta-like 1 (ITGBL1) facilitates metastatic tumor cell growth by triggering the release of pro-inflammatory cytokines IL-6 and IL-8 [63]. sEVs–miR-21 drives the activation of macrophages towards an inflammatory phenotype, leading to the production of IL-6 and inducing an inflammatory microenvironment through miR-21–TLR7–IL-6 axis [64].

In addition to the abovementioned mechanisms involved in PMN formation, other means have been described in which EVs can influence the microenvironment of remote organs. EV-associated HSPC111 promotes PMN formation and liver metastasis in mouse models. By interacting with ATP-citrate lyase (ACLY), HSPC111 impedes the lipid metabolism of CAFs, escalating levels of acetyl-CoA. In turn, elevated levels of acetyl-CoA lead to heightened CXCL5 secretion by CAFs via increased H3K27 acetylation, and CXCL5-CXCR2 axis reciprocally promotes HSPC111 release from CRC cells [65]. The expression of HuR, an RNA-binding protein, is considerably elevated in EVs derived from CRC individuals with lung metastasis compared to subjects with benign lung disease. Further, by stabilizing c-Myc mRNA expression and consequently impeding p21 expression, a cell cycle-dependent kinase inhibitor, HuR triggers proliferation, migration, and invasion of BEAS-2B bronchial epithelial cells [66].

### sEVs effects on angiogenesis

For rapidly growing malignant cells to survive and spread, angiogenesis is critical to provide them with adequate nutrients and oxygen supply [67]. Angiogenesis refers to the creation of new blood vessels, occurring when a vascular sprout arises from pre-existing blood vessels or the capillary walls bud into the vascular lumen, forming an intraluminal pillar [68]. Increasing evidence suggests that EVs play a significant role in the process of angiogenesis [69–72].

#### The effect of tumor cell-derived EVs on angiogenesis

A study reported exosomal Wnt4, originating from hypoxic CRC cells, triggers endothelial cell migration and proliferation in a hypoxia-inducible factor-1 alpha (HIF-1α)-dependent manner, thus stimulating angiogenesis and tumor growth [69]. ExomiR-1229, isolated from the serum of individuals with CRC, considerably provokes angiogenesis of human umbilical vein endothelial cells (HUVECs) in vitro and in vivo by directly targeting HIPK2 protein expression, thereby upregulating VEGF expression [70]. By suppressing FOXO1 expression, CRC-derived exosomes enriched in miR-183-5p stimulate growth, migration, and tubulogenesis of HMEC-1 cells. Besides, inhibition of miR-183-5p abolished tumor-promotive effects in nude mice [71]. A study revealed CRC-derived EVs delivering MiR-21-5p to endothelial cells, thereby facilitating angiogenesis and increasing vascular leakage. This effect is achieved by attenuating Krev interaction trapped protein 1 (KRIT1) expression, subsequently activating β-catenin signaling and upregulating angiogenesis-related genes VEGFA and cyclin D1 (Ccnd1) [72]. Haung and coauthors unveiled the underlying mechanism of tumor revascularization after withdrawal of antiangiogenic tyrosine kinase inhibitors (AA-TKIs) therapy in CRC patients. Gas6-containing EVs derived from tumor perivascular cells induce endothelial progenitor cells (EPCs) recruitment by stimulating Gas6/Axl pathway to promote the rebound effect of CRC vascularization after the AA-TKI cessation [73].

Exosome-associated transfer of miR-519a-3p from gastric cancer cells to intrahepatic macrophages induces M2 polarization of macrophages by suppressing Dual specificity protein phosphatase 2 (DUSP2) and escalating MAPK/ERK pathway, thus enhancing PMN formation and liver metastasis in gastric cancer [74]. Zhang and colleagues depicted B-cell receptor-associated protein 31 (BAP31)-overexpressing CRC cells promote angiogenesis by supporting the pro-angiogenic transformation of fibroblasts. In mechanism, exosomal miR-181a-5p derived from BAP31-overexpressing CRC cells activates residing fibroblasts by diminishing reversion-inducing cysteine-rich protein with kazal motifs (*RECK*) gene expression and subsequently amplifying MMP-9 expression and phosphorylation of Smad2/3 in CAFs [75].

Wang et al. reported adenomatous polyposis coli (APC) gene upregulates lncRNA-APC1 expression, which plays an essential tumor-suppressor role in the pathogenesis of CRC. Enforced lncRNA-APC1 expression abolishes tumor growth, metastasis, and angiogenesis through inhibition of a distinct population of exosomes, which are involved in mitogen-activated protein kinase (MAPK) and Wnt pathways. In mechanism, lncRNA-APC1 diminishes exosome production by directly binding to Rab5b mRNA and decreasing its stability [76]. Jiang et al. reported that the angiopoietin-like protein 1 (ANGPTL1) is suppressed in CRC-derived EVs, and ANGPTL1 overexpression hinders liver metastasis by dampening vascular leakiness. In mechanism, ANGPTL1 affects Kupffer cells secretion pattern via reduction of JAK2-STAT3 signaling pathway, remarkably downregulating MMP9 expression [77].

#### The effect of non-tumor cell-derived EVs on angiogenesis

TAMs play significant roles in regulating angiogenesis in various solid tumors through secreting a wide range of pro-angiogenic factors, including VEGFA, placenta growth factor (PIGF), epidermal growth factor (EGF), TGF-β, IL-1, IL-8, TNF-α, CCL2, CXCL8, CXCL12, and several matrix metalloproteinases (MMPs) [78, 79]. Recent studies indicated EVs are also implicated in TAMs-mediated tumor angiogenesis [74, 80, 81]. Yang et al. unraveled exosomal transfer of miR-155-5p and miR-221-5p from M2 macrophage to endothelial cell facilitate vascular formation in PDAC in vitro and in vivo by inhibiting transcriptional factor E2F2 [80]. A different study represented exosomes originated from M2 macrophage in PDAC are enriched in miR-501-3p, which triggers PDAC cell migration, invasion, and tubulogenesis by targeting TGFBR3 and consequently boosting TGF-β signaling [81]. CAFs can also contribute to angiogenesis in different types of malignancies through the production of distinct soluble molecules, namely VEGF, stromal cell-derived factor 1 (SDF-1), TGF-β, hepatocyte growth factor (HGF), fibroblast activation protein (FAP), and MMPs [67, 82]. Shi and colleagues reported chemoresistant CAFs accelerate CRC cell proliferation, angiogenesis, and cisplatin resistance by transferring VEGFA to malignant cells through exosomes [83].

### sEVs effects on CRC cell proliferation

It is now widely accepted that EVs promote cancer cell progression through different mechanisms, including evading growth suppressors, sustaining proliferative signaling, enabling replicative immortality, resisting cell death, inducing angiogenesis, genome instability and mutation, tumor-promoting inflammation, deregulating cellular energetics, invasion and metastasis, and avoiding immune destruction [84]. Yoshii and colleagues represented exosomes secreted from TP53-deficient colon cancer cell line HCT116, which stimulate fibroblast-mediated tumor growth by suppressing TP53 activity in fibroblasts. They identified three specific miRNAs in TP53‐deficient HCT116-derived exosomes, namely miR‐1249‐5p, miR‐6819‐5p, and miR‐6737‐5p, which play a critical role in repressing TP53 in fibroblasts and accelerating their proliferation [85]. As previously mentioned, EVs derived from CAFs reciprocally enhance cancer progression [34, 40, 45].

Teng et al. reported CRC cells selectively sort tumor suppressor miR-193a out of cells via exosomes, which are mediated by major vault protein (MVP), leading to lower levels of miR-193a in CRC cells and higher levels in plasma exosomes, thus promoting colon cancer progression and metastasis. Consistently, MVP knockout in CT26 cells causes miR-193a accumulation, subsequently inhibits CRC progression by suppressing cell cycle-related protein Caprin1 and downstream targets *cyclin D2 (CCND2)* and *c-MYC* [86]. Likewise, Liu et al. illustrated tumor suppressor miR-486-5p is notably repressed in CRC due to heightened methylation of DNA in the promoter region, resulting in elevated malignant cell growth and migration. This effect is accomplished by boosting the activity of Pleomorphic adenoma gene-like 2 (PLAGL2) and consequently amplifying insulin-like growth factor-2 (IGF-2)/β-catenin signaling [87]. A study showed that HIF-1α induces an elevation in the level of exosomal miR-361-3p level originating from hypoxic cells, which is capable of in being shuttled to CRC cells. Ultimately, exosomal miR-361-3p diminishes apoptosis and escalates CRC cell proliferation by repressing TNF receptor-associated factor 3 (TRAF3) and inducing the noncanonical NF-κB pathway [88].

By sponging miR-143, lncRNA urothelial carcinoma-associated 1 (UCA1) augments MYO6 expression in CRC cells, promoting colon cancer progression and metastasis [89]. Likewise, circIFT80 is considerably augmented both in CRC cells and their exosomes and induces CRC growth, invasion, and migration ability by acting as a sponge for miR-1236-3p and subsequently elevating HOXB7 expression [90]. Furthermore, circFMN2, a sponge for miR-1182, enhances CRC growth via positive regulation of human telomerase reverse transcriptase (hTERT) [91]. In another study, exosomal circPACRGL originated from colon cancer cells elevates the levels of TGF-β1 by sequestering miR-142-3p and miR-506-3p, thus expanding CRC growth, invasion and concomitantly inducing neutrophils differentiation toward tumor-promoting N2 neutrophils [92]. Chen et al. reported downregulation of tumor-suppressor circRHOBTB3 in CRC tissue mediated by SNF8, an ESCRT-II subcomplex interacting with specific motif in circRHOBTB3, contributes to tumor progression. Restoration of circRHOBTB3 inhibited cell proliferation and EMT by mitigating intracellular reactive oxygen species (ROS) levels and regulating metabolic enzymes, including ENO1 and ENO2 [93] (Table 1).

**Table 1:** EV cargoes associated with CRC metastasis

EV cargo	Producing cells	Expression patterns	Target cells	Related genes and/or pathways	Function/outcome	References
***Regulation of EMT***						
***Non-coding RNAs***						
miR-106b-5p	CRC cell lines	Increased	Macrophages	Triggers M2 macrophage polarization via downregulation of PDCD4 and activation of PI3Kg/AKT/mTOR signaling	Induction of EMT; facilitates liver and lung metastasis in mice	[10]
miR-21-5p and miR-155-5p	M2 macrophages	Increased	CRC cell lines	Attenuates tumor suppressor BRG1	Increased migration and invasion	[43]
miR-146a-5p	CRC cells overexpressing CXCR7	Increased	CAFs	Promotes CAF activation by attenuating ZBTB2 expression and subsequently activating NF-kB signaling	Activation of CAFs; Promotes invasion and EMT	[40]
miR-155-5p	CRC cells overexpressing CXCR7	Increased	CAFs	Promotes CAF activation and cytokines release by suppressing SOCS1 expression and inducing JAK/STAT3 signaling	Activation of CAFs; Promotes invasion and EMT	[40]
lncRNA RPPH1	CRC cell lines	Increased	CRC cells; human monocyte-derived macrophages	Binds to β-III tubulin (TUBB3) and prevents its ubiquitination in CRC cells	Promotes invasion and EMT; induction of M2 macrophage polarization	[39]
miR-92a-3p	CAFs	Increased	CRC cells	Induction of Wnt/β-catenin signaling and inhibition of mitochondrial apoptosis through downregulation of FBXW7 and MOAP1	Enhances stemness and EMT; liver metastasis; induction of chemoresistance to 5-FU/oxaliplatin	[34]
MiR-186-5p	M2 macrophage	Increased	Colon cancer cells	Inhibition of DLC1 expression and activation of the β-catenin pathway	Stimulates tumor cell growth and motility	[42]
lncRNA LINC00659	CAFs	Increased	CRC cells	Sponges miR-342-3p to increase annexin A2 (ANXA2)	Increased proliferation, invasion, migration, and EMT	[45]
***Proteins***						
FZD10	colon cancer cell lines	Increased	Colonic epithelial cells	Induction of Wnt/β-catenin signaling	Promotes EMT; enhances cancer cell viability and proliferation	[37, 38]
Wnt	Macrophages	Increased	NA	Activation of Wnt/β-catenin	Maintenance of intestinal stem cells; increased intestinal repair after radiation in mice	[35]
***Premetastatic niche formation***						
*** Non-coding RNAs***						
miR-25-3p	CRC cell lines	Increased	Human umbilical vein endothelial cells (HUVEC)	Increases expression of VEGFR2 and reduces tight junction proteins ZO-1, occluding, and Claudin5 by silencing KLF2 and KLF4	Increased vascular permeability; angiogenesis; liver and lung metastasis in mice	[48]
miR-320c	Blood sample of metastatic CRC patients; CRC cell lines	Increased	NA	NA	Promotes mesenchymal-epithelial transition (MET) in metastasized cells; formation of PMN	[52]
miR-934	CRC cells	Increased	Macrophages; human Kupffer cells	Promotes M2 macrophage polarization by attenuating PTEN and inducing PI3K/AKT signaling; M2 macrophages facilitate liver metastasis through CXCL13/CXCR5 axis	M2 macrophage polarization; liver metastasis	[53]
miR-181a-5p	highly metastatic CRC cells	Increased	Hepatic stellate cells (HSCs)	Activation of HSCs by downregulating SOCS3 and inducing IL6/STAT3 signaling	Liver metastasis	[51]
miR-21	CRC cells	Increased	Liver macrophages; human macrophage cell line	Activates Toll-like receptor 7 (TLR7) in liver macrophages	Macrophage polarization toward IL-6 secreting phenotype; induction of inflammatory PMN in the liver	[64]
miR-135a-5p	CRC cells; Serum of CRC patients; tumor tissues from CRC patients	Increased	Kupffer cells	Impedes tumor suppressor kinase LATS2 expression, and subsequently activates YAP1/TEAD1 complex and elevates MMP7 expression	mice liver metastasis; inhibition of CD30-mediated CD4 + T cells activation; Increased cell adhesions; PMN formation	[54]
***Proteins***						
Integrin beta-like 1 (ITGBL1)	Tumor tissues from CRC patients; plasma sample of CRC patients; CRC cell lines	Increased	Hepatic fibroblasts; hepatic stellate cells	Binds to TNFAIP3 and triggers the NF-κB signaling pathway	Fibroblast activation; increased secretion of proinflammatory cytokines; PMN formation	[63]
RNA binding protein HuR	CRC cell line	Increased	Bronchial epithelial cells	Stabilizes c-Myc and downregulates p21 expression	Increased proliferation, migration, and invasion of bronchial cells	[66]
Interferon regulatory factor 2 (IRF2)	Mouse colon carcinoma cells; blood samples of CRC patients	Increased	Macrophages	Increased secretion of VEGF-C from macrophages	Lymph node metastasis	[49]
TGF-β1	CRC cells	Increased	Hepatic stellate cells (HSCs)	Provokes HSCs differentiation into CAFs via Activation of HSCs chemokines signaling; Utilizes MDSCs into the liver; abrogates NK cell cytotoxicity by repressing NKG2D	Facilitates premetastatic immunosuppressive niche in the liver; inhibition of NK cell cytotoxicity; induction of HSCs differentiation into CAFs	[55]
HSPC111	CRC cell lines	Increased	HSCs cell line	Education of HSCs into CAFs; interacts with ATP-citrate lyase (ACLY), resulting in accumulation of acetyl-CoA in CAFs; promotes CXCL5 secretion by increasing H3K27 acetylation	PMN formation and liver metastasis; impedes the lipid metabolism of CAFs;	[65]
***Angiogenesis***						
**Non-coding RNAs**						
miR-1229	Tumor tissues and blood samples from CRC patients; CRC cell lines	Increased	HUVECs	Inhibition of HIPK2 protein expression; promotes MEF2C-mediated activation of VEGF	Facilitates proliferation, migration, and tube formation of HUVECs	[70]
miR-183-5p	CRC cell lines	Increased	Human endothelial cells (HMEC-1)	Decreased FOXO1 expression	Facilitates proliferation, migration, and tubulogenesis of HMEC-1	[71]
miR-21-5p	CRC cells	Increased	HUVECs	Reduced Krev interaction trapped protein 1 (KRIT1) expression; increased activation of the β-catenin signaling pathway, and upregulation of downstream VEGFa and Ccnd1	Angiogenesis; increased vascular permeability	[72]
miR-181a-5p	BAP31-overexpressing CRC cells	Increased	human lung normal fibroblast cells; mouse embryonic fibroblast cells	Silences reversion-inducing cysteine-rich protein with kazal motifs (RECK); upregulation of MMP-9 and phosphorylation of Smad2/3	Differentiation of fibroblasts into proangiogenic CAFs; angiogenesis	[75]
***Proteins***						
Wnt4	Hypoxic CRC cells	Increased	HUVEC	Wnt/β-catenin signaling	Promotes tumor growth and angiogenesis in vitro and in vivo	[69]
Gas6	Tumor perivascular cells	Increased	Endothelial progenitor cells	Employs endothelial progenitor cells through activation of the Axl pathway	Instigates cancer revascularization after antiangiogenic therapy withdrawal	[73]
VEGFA	Chemoresistant CAFs	Increased	CRC cells	VEGFA	Provokes proliferation, cisplatin resistance, and angiogenesis of CRC	[83]
Angiopoietin-like protein 1 (ANGPTL1)	CRC cell line	Decreased	Mouse Kupffer cell line	Downregulates MMP9 expression in KCs via inhibition of JAK2-STAT3 signaling	Attenuates vascular leakiness and hinders liver metastasis	[77]
***Tumor cells proliferation***						
miR-6819-5p, miR-6737-5p, and miR-1249-5p	TP53 mutant colon cancer cells	Increased	Human colon fibroblasts; human lung fibroblasts	Decreased TP53 expression in fibroblasts	Instigates fibroblast‐mediated tumor growth; enhances fibroblast proliferation	[85]
miR-193a	Mouse colon carcinoma cells; human colon cancer cells	Increased in serum exosomes; decreased in CRC cells	NA	Sorting of tumor-suppressor miR-193a out of cells leads to tumor growth through; inhibition of Caprin1 and downstream targets cyclin D2 and c-MYC	Inhibits tumor progression	[86]
miR-486-5p	CRC tissues and cell lines; Plasma specimens from CRC patients	Increased in serum exosomes; decreased in CRC cells	NA	Exosomal packaging of tumor-suppressor miR-486-5p out of cells;downregulation of PLAGL2 and downstream β-catenin and IGF2 signaling	Inhibits proliferation and migration in vitro and in vivo	[87]
miR-361-3p	Hypoxic CRC cells	Increased	CRC cells	Targets TNF receptor-associated factor 3 (TRAF3) and promotes noncanonical NF‐κB signaling	Decreased apoptosis and increased proliferation	[88]
lncRNA UCA1	CRC tissues and cell lines; blood samples from CRC patients	Increased	CRC cells	Sponges miR-143 and escalates MYO6 expression	Accelerates proliferation and migration in vitro and in vivo	[89]
circIFT80	CRC tissues and cell lines; blood samples from CRC patients	Increased	CRC cells	Sponges miR-1236-3p and escalates HOXB7 expression	Instigates proliferation, migration, and EMT	[90]
circFMN2	CRC cells; CRC tissues; blood samples from CRC patients	Increased	CRC cells	Acts as a sponge for miR-1182 and elevates hTERT expression	Increased proliferation and migration	[91]
circPACRGL	CRC cells	Increased	CRC cells; polymorphonuclear neutrophils	Sponges miR-142-3p and miR-506-3p and elevates TGF-β1 expression	Increased progression and migration; N2 neutrophil differentiation	[92]
circRHOBTB3	CRC cell lines; tissues and sera of CRC patients	Increased in serum exosomes; decreased in CRC tissues	NA	Excretion of tumor-suppressor circRHOBTB3 out of cells; regulation of intracellular ROS levels and metabolic enzymes ENO1 and ENO2	Inhibition of CRC cell proliferation and EMT in vitro and in vivo	[93]

## sEVs regulate immune response in CRC

In the primary and premetastatic tumor site, the surrounding environment, including cancer cells, stromal cells, immune components, signaling molecules, extracellular matrix, and adjacent blood vessels, known as the tumor microenvironment (TME), can either profoundly contribute to or suppress malignant progression [94, 95]. Typically, immune surveillance hinders tumor cell growth; thus, CRC cell expansion and metastasis are extensively linked to their ability to modify TME and conduct an immune-suppressive environment [96]. By orchestrating cell–cell communication in TME, EVs may impair anti-tumor response and enhance immune editing by driving stromal and immune cells transformation toward tumor-promoting cells such as CAFs, TAMs, Tregs, TANs, and MDSCs [97].

Macrophages are essential in tissue homeostasis, maintaining the innate immune response, and inflammation [98]. Macrophages can evolve through specific differentiation into two phenotypic polarizations: proinflammatory or classically activated M1 phenotype and anti-inflammatory or alternatively activated M2 phenotype [99]. M1 macrophages, stimulated by cytokines such as IFN-γ, lipopolysaccharide (LPS), and TNF-α, elicit a robust inflammatory response and secrete proinflammatory cytokines such as IL-6, IL-12, IFN-γ, TNF-α, and ROS [100, 101]. In contrast, in the presence of factors such as IL-4, IL-13, IL-10, or glucocorticoids, M2 macrophages produce IL-10 and TGF-β, acting in tissue remodeling, angiogenesis, and tumor progression [102]. A study showed that mutant TP53 CRC cells promote macrophage differentiation towards M2 macrophage with increased TGF-β production in a paracrine manner by secreting exosomal miR-1246 [103]. Additionally, miR-155-5p derived from M2-macrophage triggers SW48 cells proliferation, antiapoptotic ability, and immune evasion by targeting zinc-finger-type-containing 12B (ZC3H12B) expression and elevating IL-6 production upon internalization by SW48 cells [104]. Another study indicated lncRNA RPPH1 uptake by macrophages facilitates a shift from M1 phenotype to M2 TAMs, thereby promoting CRC cell proliferation and metastasis in the animal model [39].

Exosomes originating from CRC cells can shuttle TGF-β to T cells, initiating TGF-β/Smad signaling and suppressing stress-activated protein kinase (SAPK) signaling. This drives the transformation of T cells into a Treg-like phenotype, thus escalating tumor growth [105]. CRC cells possess the ability to trigger apoptosis in CD8 + T cells through exosomes harboring proapoptotic molecules Fas ligand and tumor necrosis factor-related apoptosis inducing ligand (TRAIL) [106]. Poggio et al. have depicted exosomes from various cancers, including CRC, carry PD-L1 on their surface. PD-L1 can systematically bind to its receptor PD-1 on T cells in the regional lymph nodes, resulting in the inhibition of T cells. Interestingly, removal of exosomal PD-L1 combined with PD-L1 blockade antibodies remarkably synergize to abolish malignant progression and remote metastasis, even in anti-PD-L1 resistance models [107]. Exosomal miR-424, released by CRC cells, can be internalized by T cells and DCs present within the tumor microenvironment. Once internalized, it exerts a negative regulatory effect on CD28, CD80, and CD86 costimulatory molecules, inducing resistance to immune checkpoint blockade treatment. Simultaneously, miR-424 depletion promotes adaptive anti-tumor immunity and increases advanced tumor sensitivity to immune checkpoint blockade therapy [108]. A study unraveled that by shuttling miR-21-5p and miR-200a via EVs, CRC cells augment PD-L1 levels in M2 macrophages by suppressing PTEN and SOCS1 expression and upregulating AKT and STAT1 expression, resulting in M2 suppressive activity on CD8 + T cells [109]. Moreover, by targeting Numb, CSCs-derived exosomal miR-146a-5p promotes CRC cell stemness, and elevated concentrations of miR-146a-5p in sera correlate with increased CD66 + neutrophils infiltration and reduced counts of CD8 + T cells in TME [110].

Neutrophils, being the predominant leukocytes, are crucial for the body’s protection against pathogens and microbial infection. As active participants in TME, they can differentiate into N2-like neutrophils and assist in tumor growth [111]. By amplifying NF-κB signaling and subsequently escalating IL-1β expression, CSCs-derived exosomes extend neutrophils survival in the bone marrow via the transfer of triphosphate RNAs. Therefore, secretion of CSCs chemokines attracts exosome-trained neutrophils to the primary tumor, which increases CRC tumorigenicity [112]. Furthermore, exosome-associated miR-4780 isolated from N2-like neutrophil exacerbates EMT and tubulogenesis in COLO205 and SW480 cell lines by repressing its downstream target SOX11 [111]. The uptake of tumor-derived EVs harboring oncogenic *HRAS* gene by neutrophils enhances IL-6 production and tissue factor activation in xenograft mice, resulting in a proinflammatory response [113]. Neutrophils have the capability to capture and sustain cancer cells in remote organs by elaborating mesh-like structures containing strands of DNA and active peptides, so-called neutrophil extracellular traps (NETs). Although NETs represent a significant role in defense against infection under normal conditions, they are also implicated in cancer progression. EVs are engaged in neutrophil accumulation and NET deposition in lymphatic nodes through accelerating neutrophil infiltration by inducing CXCL8/2 secretion from lymphatic endothelial cells. Furthermore, Rab27a knockdown attenuates neutrophil recruitment, NETs formation, and lymph node metastasis. It is noteworthy to mention that disruption of NETs through neutrophil depletion diminishes lymph node metastasis [114].

CAFs constitute a prominent component of TME with a pro-tumorigenic capacity that plays essential roles in tumor growth, metastasis, ECM remodeling, immune suppression, and chemotherapy resistance [115]. CAFs can be distinct from resting fibroblasts based on their contractile characteristic and expression of several CAF markers, such as alpha-smooth muscle actin (α-SMA) and fibroblast-activation protein (FAP) [116]. They can develop from diverse cell precursors, which results in their heterogeneity [115]. Oxidative stress, hypoxia, several molecules including TGF-β, IL-1, IL-6, Lysophosphatidic acid, fibroblast growth factor type 2 (FGF-2), epidermal growth factor (EGF), and platelet-derived growth factor (PDGF) have been shown to mediate employment and stimulation of CAFs [116, 117]. A study represented EVs harboring miR-10b, originating from CRC cells, attenuate fibroblast proliferation while provoking TGF-β and α-SMA expression and enhancing CAFs activation. Mechanistically, miR-10b abolishes the expression of PIK3CA, downregulates PI3K/Akt/mTOR pathway, and activated fibroblasts contribute to CRC growth [118]. CAFs-derived EVs facilitate progression and metastasis of CRC through multiple mechanisms. Studies demonstrated CAFs-derived EVs are involved in angiogenesis, migration, invasion, acquisition of stem-like features, and chemotherapy resistance in CRC [34, 40, 75].

Additionally, the involvement of additional immune cells, including DCs and MDSCs, has been suggested in CRC development [119, 120]. Tumor-derived EVs dampen CD14 + monocytes differentiation into DCs and trigger the production of monocytes, which secrete TGF-β and possess inhibitory effects on T cells. Mechanistically, EVs affect CD14 + monocytes by downregulating CD80 and CD86 costimulatory molecules and attenuating HLA class II expression [119]. Wang and colleagues explored the role of MDSCs in CRC progression and reported exosomal S100A9 from MDSCs can be internalized by CT-26 cells, which simultaneously intensifies CT-26 cell stemness and recruitment of MDSCs resulting in decreased anti-tumor immunity [120].

Despite the wealth of research indicating the pro-tumorigenic effects of EVs derived from metastatic tumors, Plebanek et al. demonstrated EVs originating from poorly metastatic melanomas diminish lung metastasis by improving immune surveillance through patrolling monocytes and NK cells. Non-metastatic EVs display this anti-metastatic function by promoting macrophage polarization and killing ability through harboring pigment epithelium-derived factor (PEDF) on their outer surface [121]. CRC-derived EVs carrying HSP70 on their surface, prime NK cells by boosting their motility and cytolytic potential. This is mediated by secretion of granzyme B, which leads to the acceleration of apoptosis in cancer cells [122]. In another study, exosomes containing HSP70 originated from heat-stressed colon cancer cells, abolished TGF-β1-induced Treg differentiation, and enhanced Th17 generation in the animal model by boosting IL-6 production in BMDCs, thereby improving anti-tumor response [123] (Table 2).

**Table 2:** Regulatory mechanism of EV cargoes in the development of immune suppression in CRC

EV cargo	Producing cells	Expression patterns	Target cells	Related genes and/or pathways	Function/outcome	References
*** Non-coding RNAs***						
miR-1246	mutp53 CRC cells	Increased	Primary monocytes	NA	Macrophage reprogramming into tumor-promoting phenotype with increased TGF-β secretion	[103]
miR-155-5p	M2 macrophage	Increased	Colon cancer cells	Inhibition of ZC3H12B expression and upregulation of IL-6; escalates CD3 + T cell proliferation and the proportion of IFN-γ + T cells	Increased cancer cell proliferation and antiapoptosis ability; abrogates T cell immune response and promotes tumor formation	[104]
miR-424	Human CRC cell lines; mouse CRC cell lines	Increased	Tumor-infiltrating T cells and DCs	Diminishes CD28-CD80/86 costimulatory pathway	Resistance to immune checkpoint blockade immunotherapy; dampens T cell antitumor response	[108]
miR-21-5p and miR-200a	human CRC cells; murine colon cancer cells	Increased	Human monocyte cell line	Induces M2 polarization and PD‐L1 expression in macrophages through regulation of PTEN/AKT and SCOC1/STAT1 pathways	M2 macrophage polarization; suppresses CD8 + T cell activity and enhances tumor growth	[109]
miR-146a-5p	CRC stem cells	Increased	CRC cells	attenuates Numb expression; increased tumor-infiltrating CD66b + neutrophils and decreased number of CD8 + T cells	Increased stem-like properties and tumorigenesis; establishes immunosuppressive TME	[110]
miR-4780	N2-like neutrophils	Increased	CRC cells	Targets SOX11 expressions	Increased viability, migration, and invasion; promotes EMT and angiogenesis	[111]
miR-10b	CRC cell line; tissue specimens from CRC patients	Increased	Human fibroblasts	Augments TGF-β and SM α-actin expression by suppressing PIK3CA expression and PI3K/Akt/mTOR pathway	Promotes fibroblast activation and tumor growth	[118]
***Proteins***						
TGF-β1	colon cancer cells	Increased	Human T-cell leukemia Jurkat cells, PBMCs, and CD4 + T cells	Triggers TGF-β/Smad signaling and diminishes SAPK signaling	Inhibition of T cell proliferation; transforms T cells into Treg-like phenotype; increased tumor growth	[105]
TNF-related apoptosis-inducing ligand (TRAIL) and Fas ligand	CRC cell lines; blood sample of CRC patients	Increased	CD8 + T cells from CRC patients	Fas ligand and TRAIL	Induction of apoptosis in CD8 + T cells in vivo and in vitro	[106]
PD-L1	Cancer cell lines	Increased	T cells	PD1/PD-L1 pathway	Inhibition of T cell activity in lymph nodes; increased tumor growth	[107]
S100A9	Granulocytic MDSCs (G-MDSCs)	Increased	CT‐26 colon cancer cells	Activation of NF‐κB and STAT3 signaling	Increased stemness and growth in tumor-bearing mice; increased susceptibility to colitis‐associated colon cancer in mice; enhanced recruitment of MDSCs	[120]
*** Other cargoes***						
Triphosphate RNAs	Murine CRC stem cells	Increased	Bone marrow-derived neutrophils	Upregulates NF-κB signaling and induces IL-1β expression	Extends neutrophil survival in the bone marrow; induction of a pro-tumoral phenotype in neutrophils; increased tumorigenicity	[112]
Tumor-derived EVs	CRC cell lines; melanoma cell lines	NA	CD14 + monocytes	Downregulation of CD80 and CD86 costimulatory molecules and HLA class II expression	Hinders CD14 + monocytes differentiation into DCs; increased TGF-B-secreting monocytes with suppressive activity on T cells	[119]

## Double-faceted role of autophagy in CRC carcinogenesis and its interaction with sEVs

Autophagy is a well-preserved physiological mechanism through which cells capture and break down damaged proteins and organelles [11]. Three distinct forms of autophagy exist: microautophagy, chaperone-mediated autophagy (CMA), and macroautophagy. Despite common features, they can be distinguished based on their cargo selection and the specific mechanism they utilize for delivering their cargo to the lysosomes [124]. Macroautophagy is the best-characterized and most prevalent form of autophagy. It initiates with the nucleation of the isolation membrane or phagophore. Phagophore expands and progressively engulfs cytoplasmic components, eventually maturing into an autophagosome. Following that, the autophagosome combines with the lysosome, leading to the creation of an autolysosome where the engulfed contents undergo degradation [124, 125]. Environmental stressors such as oxygen deprivation, nutrient deficiency, and DNA damage elevate the basal level of autophagy in cells [126]. Autophagy exhibits a dual function in cancer. It serves as a tumor-suppressor during the initial stages of tumor development, impeding malignant cell proliferation, whereas it supports tumor growth in later stages of tumorigenesis [127, 128].

### Double-faceted role of autophagy in CRC

Autophagy was initially considered to abolish tumor progression by removing detrimental substances and impaired organelles, thereby preventing the accumulation of genetic abnormalities [129]. Early indications of autophagy's capability to suppress tumor growth emerged from research focused on the beclin-1 (*BECN1*) gene. Initial reports demonstrate a significant incidence of monoallelic deletion of the *BECN1* gene in breast, prostate, and ovarian cancer. Furthermore, the depletion of *BECN1* in cancer cells and animal models contributes to a repressed autophagy flux and accelerated malignant growth [130–133]. Later studies revealed *BECN1* is adjacent to breast cancer 1 (*BRCA1*) on chromosome 17q21, and *BECN1* deletion does not occur independently of *BRCA1*, indicating *BRCA1* loss is the primary mutation responsible for breast cancer development [134, 135]. Moreover, further studies have represented that the deletion of additional crucial autophagy-related genes (ATGs) represses tumor development in cancer. The ultraviolet irradiation resistance-associated gene (*UVRAG*), associated with *BECN1*, positively regulates autophagy, and mutated UVRAG promotes tumorigenesis in CRC [136]. Similarly, depletion of Bax-interacting factor-1 (BIF-1), which is also related to beclin-1, results in malignant progression in gastric cancer and CRC [137–139].

Common oncogenic alterations in PI3K, AKT, and PTEN can activate the mechanistic target of rapamycin (mTOR), thus inhibiting autophagy and enhancing tumorigenesis, while tumor suppressors activate autophagy by negatively regulating mTOR and AMPK [128, 140, 141]. Chromatin immunoprecipitation sequencing has unraveled distinct autophagy genes that are directly targeted by P53, highlighting the involvement of autophagy in apoptosis and tumor suppression regulated by p53 [142]. In contrast to nuclear P53, cytoplasmic p53 can repress autophagy at the basal level through protein–protein interaction with the autophagic machinery [143]. On the other hand, heightened cytoplasmic levels of P53 resulting from cellular stressors, such as DNA damage, stimulate the initiation of autophagy by provoking the expression of *DRAM1* [144].

Autophagy also regulates ROS production, thus diminishing tumor generation [145]. The initiation of tumorigenesis in epithelial cells triggered by Ras is abolished by autophagy, which restricts ROS production [146]. Additionally, by preventing inflammation and necrosis, autophagy may suppress cancer cell proliferation [127]. The *ATG16L1* Thr300Ala polymorphism is connected to a heightened susceptibility to Crohn's disease. Lassen and coauthors demonstrated *ATG16L1* Thr300Ala knock-in mice confer morphological abnormalities in Paneth and goblet cells associated with Crohn's disease. Interestingly, *ATG16L1* Thr300Ala exhibits only a slight disturbance in the baseline autophagy but exerts a more pronounced defect in antibacterial autophagy and increased IL-1β secretion in embryonic fibroblasts [147].

While insufficient autophagy can contribute to the onset of tumors, an elevated level of autophagy also facilitates the advancement toward invasive malignancies [128, 148]. As a result of heightened cellular proliferation, tumor cells experience elevated oxygen and metabolic requirements. In response to hypoxia and nutrient deprivation, autophagy is triggered to ensure cell viability [128]. Metabolic stress is observed in cells with impaired autophagy and reduced cell survival in vivo [128]. By activating stress response signaling pathways, autophagy enables tumor cells to survive and adapt under hypoxia [149]. The hypoxic regions of the tumor exhibit an acceleration in the level of autophagy [150]. HIF-1α escalates autophagy flux and modulates its target genes under low oxygen conditions [149, 151].

Cancer stem cells (CSCs) have higher levels of autophagy than other tumor cells, enabling them to maintain their stemness and survive under drastic conditions in the tumor microenvironment [152, 153]. Downregulation of ATG4A attenuates CSC-like properties in breast cancer, indicating the importance of autophagy in sustaining CSC characteristics [153]. Moreover, autophagy triggered by starvation initiates EMT and invasion in hepatocellular carcinoma (HCC) cells through TGF-β/Smad3 signaling, and inhibition of autophagy by depleting Atg3 or Atg7 abolishes this effect [154]. In order to survive after detachment from ECM, tumor cells undergoing EMT need to survive detachment-induced apoptosis, an apoptotic process known as anoikis [155]. An in vivo model of HCC portrayed that autophagy inhibition eradicated the ability to evade anoikis and hindered lung metastasis through the regulation of apoptotic signaling [156]. It was demonstrated that using an autophagy inhibitor, chloroquine diphosphate, improved the effect of 5-FU on colon cancer cells, suggesting autophagy potentiates resistance to chemotherapy [157].

### The crosstalk between autophagy and sEVs in CRC

Autophagy and EV release are both elevated in tumor cells as a part of cell response to stressful conditions to promote tumor cell survival [128, 158]. They both involve vesicular trafficking and share common proteins, complexes, and pathways. Despite limited knowledge regarding the specific molecular mechanism governing the interaction between autophagy and EVs, recent studies have placed significant emphasis on investigating whether their combined activation in malignant cells synergistically contributes to malignant progression [159].

Autophagy displays its pivotal role through canonical pathway (degradative function) and non-canonical pathway (secretory function). The latter represents the secretory mechanism of autophagy, known as secretory autophagy (SA). SA facilitates the unconventional secretion of cytosolic proteins lacking signal peptide and thus cannot be secreted through conventional pathways via ER and Golgi apparatus [160–162]. Similar to degradative autophagy, SA involves ATG families and ESCRT proteins [163]. Furthermore, several components of vesicle trafficking, including Rab-GTPases and SNAREs, have been implicated in SA and guide autophagosomes to merge with the cell membrane [164, 165]. The interconnection linking autophagy with the generation and secretion of EV has been well-established in the context of amphisome formation [12]. Once MVBs are formed, they can have two fates: First, in the presence of autophagy stimuli, autophagosome fuses with MVB to form amphisome, which in turn combines with lysosome for degradation [12, 166]. Therefore, amphisome formation negatively regulates EV release by preventing the merging of MVB with the plasma membrane. Second, when autophagy is inhibited, MVBs can freely merge with the plasma membrane and secrete EVs into the surrounding extracellular environment [21]. Amphisomes were initially thought of as exclusively degradative organelles. Recently, they were also implicated in non-degradative mechanisms [167, 168]. For instance, autophagy protein light chain 3beta (LC3β) is colocalized with early endosomal marker EEA1 in mice colonic goblet cells, indicating the fusion of autophagosomes and endosomes for efficient mucus secretion [167]. Likewise, exosomal secretion of ANXA2 in lung epithelial cells elicited by IFN-γ is associated with autophagosomes fusion with MVBs, followed by the merging of amphisomes with PM [168].

A growing body of studies described common proteins and complexes operating in both autophagy and EV pathways. Rab11 governs the fusion of autophagosomes and MVBs in the autophagy-mediated exosomal secretion of ANXA2. Additionally, Rab8A and Rab27A guide amphisomes harboring ANXA2 towards PM for secretion [168]. Murrow et al. reported that by interacting with ESCRT-III-associated protein Alg-2 interacting protein-X (ALIX), ATG12-ATG3 complex regulates multiple processes, including autophagosome maturation, endosomal trafficking, exosome generation, and viral budding. ALIX or ATG12-ATG3 knockdown diminishes autophagy flux and exosome biogenesis. Strikingly, ALIX or ATG12-ATG3 depletion does not impact autophagy triggered by starvation, suggesting nutrition deprivation bypasses the requirement of these molecules in autolysosome formation [169]. Similarly, ATG16L1 and ATG5 regulate exosome production. Mechanistically, ATG16L1 and ATG5 attenuate acidification of MVBs by impairing the function of vacuolar proton pumps V1/V0 –ATPase at MVBs, thereby avoiding lysosomal degradation and promoting exosomes release by enhancing MVBs fusion with the plasma membrane. Moreover, ATG5-ATG16L1 complex escalates breast cancer cell motility and metastasis by controlling exosome release [170, 171]. Bader and colleagues illustrated transmembrane ATG9 is essential for the ILVs formation in *Drosophila* since depletion of ATG9 represses autophagy flux and ILVs generation in amphisome. Nevertheless, it remains ambiguous if these intraluminal vesicles (ILVs) are released in the form of exosomes [172]. R-SNARE Sec22b is reported to interact with TRIM16, a secretory autophagy receptor, and their interaction is mandatory for IL‐1β secretion upon lysosomal damage in THP‐1 cell line and primary macrophages. In mechanism, SA utilizes the specialized cytosolic cargo receptor TRIM16 and R-SNARE Sec22b in cooperation with Q-SNAREs syntaxin 3, syntaxin 4, SNAP-23, and SNAP-29 for cargo secretion [165]. A study confirmed that in an imatinib-resistant chronic myeloid leukemia (CML) cell line, dasatinib improved apoptosis through diminishing Akt/mTOR activities while abolished exosome secretion and autophagy by attenuating beclin 1 and Vps34 expression [173]. Vps34, a PI3/PI4-kinase family member, has been shown to regulate mTOR pathway and interact with beclin 1, promoting autophagy [174]. Notably, inhibition of mechanistic target of rapamycin complex 1 (mTORC1) by rapamycin showed no effect on exosome secretion and autophagic flux in imatinib-resistant CML cells [173]. Leidal and colleagues reported that LC3-conjugation machinery is essential in EV cargo packaging and secretion of RNA-binding proteins (RBPs) and small non-coding RNAs. They introduced LC3B-dependent EV loading and secretion (LDELS) as a regulatory mechanism, which employs neutral sphingomyelinase 2 (nSMase2) and Factor-associated with nSMase2 (FAN) to specify extracellular RNA loading into EV efficiently [175].

Specific exosomal non-coding RNAs (ncRNAs) released by tumor cells seem crucial in regulating autophagy activity in recipient cells [176, 177]. Pan et al. unveiled that circATG4B is remarkably enriched in oxaliplatin-resistant CRC cells-derived EVs, which are capable of being shuttled to sensitive cells, triggering oxaliplatin resistance by promoting autophagy. While circRNAs commonly function as sponges for miRNAs, some circRNA have been shown to encode novel proteins. circATG4B encodes the circATG4B-222aa protein that targets transmembrane p24-trafficking protein 10 (TMED10) by competing with ATG4B, thereby hampering TMED10-mediated ATG4B inhibition and increasing autophagy [176]. By releasing exo-miR-4534, P53-deficient CRC cells promote fibroblast activation by inhibiting autophagy in fibroblasts through dampening ATG2B expression [177]. Yeon and colleagues investigated the mechanism of the cancer/testis antigen CAGE in tumorigenesis. They found CT26 mouse colon cancer cells overexpressing CAGE augment autophagy flux by suppressing miR-140-5p and subsequently upregulating Wnt1 expression. Additionally, CAGE also upregulates Wnt1 expression at the transcriptional level. Intriguingly, CT26 cells overexpressing CAGE release exosomes abundant in Wnt1, thereby escalating autophagy flux in mast cells and macrophages [178].

## Diagnostic and prognostic implication of sEVs in CRC

sEVs potential to serve as diagnostic and prognostic biomarkers in cancer is attributed to their significant roles in cancer development and metastasis, their stability and reproducibility, their ability to reflect the tumor cell state, and their non-invasive detection in plasma or urine from liquid biopsies. Although sEVs are holding promises as highly sensitive and specific biomarkers, the characterization of such heterogenous and small molecules remains challenging [179].

### sEVs non-coding RNAs as CRC diagnostic and prognostic biomarkers

Transcriptomics research unraveled EVs ncRNA cargoes constitute essential biomarkers for CRC diagnosis. A study showed seven exosomal miRNAs, including miR-21, let-7a, miR-1246miR-1229, miR-150, miR-223, and miR-23a, portray significant expression in the serum of primary CRC subjects in an early stage of tumor compared with healthy controls, and exhibited a remarkable downregulation following resection of tumors [179]. The expression of exosomal miR-27a and miR-130a are substantially elevated in CRC. Notably, miR-27a and miR-130a contribute to CRC progression through upregulation of Wnt/β-catenin and TGF-β pathway, and their heightened expression is linked to an unfavorable prognosis [180]. miR-125a-3p and miR-320c are highly enriched in exosomes derived from CRC patients than their corresponding controls and can be employed as diagnostic markers. The coexistence of miR-125a-3p and carcinoembryonic antigen (CEA) amplifies the diagnostic capability of CEA in identifying early-stage colon cancer [181]. The heightened concentration of miR-122 in serum exosomes can distinguish CRC individuals with liver metastasis from healthy donors and CRC subjects without LM [182]. A study showed miR-193a and let-7 g expression in peritoneal metastatic CRC cells in comparison with primary CRC cells derived from the same patients were downregulated and upregulated, respectively [183]. Augmented concentration of miR-23a and miR-301a has been proposed to discriminate between CRC subjects and tumor-free corresponding but did not correlate with clinicopathologic features of patients [184]. The concentration of miR-99b-5p and miR-150-5p is attenuated in serum exosomes of CRC individuals as opposed to subjects without the disease and those with benign disease, and it recovers after surgery [185]. circLPAR1 is strongly suppressed in CRC, but it arises following surgery, and this downregulation negatively correlates with overall survival. Mechanistically, circLPAR1 diminishes expression of the oncogene bromodomain-containing protein 4 (*BRD4*) by sponging eukaryotic translation initiation factor 3 subunit h (eIF3h) and preventing the interaction between methyltransferase-like 3 (METTL3) and eIF3h [186]. Wei et al. demonstrated that decreased plasma concentrations of EV-miR-193a-5p can serve as a discriminatory factor between CRC patients, individuals with colorectal adenoma, and healthy groups and are associated with worse overall survival. They also found that miR-193a-5p abolishes the migratory ability of CRC cells by repressing the expression of CUT-like homeobox 1 (*CUX1*) and intersectin 1 (*ITSN1*), which play pivotal roles in regulating EMT [187]. circ-PNN and hsa-circ-0004771 exhibit elevated concentrations in serum exosomes of CRC individuals, making them potential novel biomarkers for timely detection of the disease [188, 189]. Hu and coauthors identified a group of six lncRNAs, LNCV6_116109, LNCV6_98390, LNCV_108266, LNCV6_38772, LNCV6_84003, and LNCV6_98602, that exhibit higher expression in CRC, specifically in stage I and II of the disease. These lncRNAs have the capability to diagnose CRC before progression to an advanced stage [190]. Aberrant expression of LINC02418 can indicate CRC and enhance cancer cell proliferation by acting as a competing endogenous RNA (ceRNA), thereby boosting the activation of MELK by inhibiting miR-1273 g-3p [191].

CRC patients exhibit a lower concentration of exosomal miR‐548c‐5p in serum compared to healthy subjects. This reduced expression is more significant in liver metastasis and advanced stage of disease and is accompanied by attenuated overall survival in patients [192]. Exosomal miR-6803-5p is observed to be remarkably upregulated and predicts poor outcomes in CRC [193]. Liu and coauthors represented lncRNA GAS5 is highly expressed in CRC, while miR-221 exerts lower concentration in tissue, plasma, and exosomes. Both lncRNA GAS5 and miR-221 reflect clinicopathological features of patients in a stage-dependent fashion [194]. Heightened levels of lncRNA 91H in CRC trigger migratory capacity and progression of malignant cells by modifying the expression of heterogeneous nuclear ribonucleoprotein K (HNRNPK) and is accompanied by an increased risk of recurrence and metastasis [195]. Another study depicted that an attenuated level of *HOTTIP* in CRC-derived exosomes is accompanied by unfavorable overall survival, which can independently predict post-surgical survival [196].

### sEVs protein and lipid profiles as CRC diagnostic and prognostic biomarkers

Aside from ncRNAs, EV protein and lipid profiles may exert potential diagnostic implications in CRC, while lipidome analysis of EVs did not provide a clear diagnostic implication. For instance, a study demonstrated fatty acid saturation attenuated and shifted from 34:1 phosphatidylcholine (PC), phosphatidylethanolamine (PE), and phosphatidylinositol (PI) in non-affected individuals to 38:4 species in subjects with pathological changes in the colon [197]. Another study reported exosomes from non-metastatic cell lines and primary CRC patients exhibit remarkable increase in PC 34:1, sphingomyelin (SM) d18:1/16:0, PE 36:2, hexosylceramide (HexCer) d18:1/24:0, and HexCer d18:1/24:1 when contrasted with healthy controls, while same lipids were attenuated in metastatic cell lines and patients [198].

Exosomes derived from MDSCs express augmented levels of S100A9 in CRC relative to healthy controls, and aberrant expression of S100A9 can be predictive for cancer development and relapse [120]. CRC-derived exosomes express elevated levels of Glypican-1 (GPC1), and GPC1 level attenuates after surgical treatment [199]. Moreover, exosomal copine III (CPNE3) acts as an independent prognostic and diagnostic marker since it is overexpressed in CRC and negatively correlates with survival outcomes [200]. Further, CRC-derived exosomes display augmented levels of cytokeratin 19 (CK19), tumor-associated glycoprotein 72 (TAG72), and cancer antigen 125 (CA125). TAG72 is mainly expressed in exosomes originating from CRC cells that exhibit resistance to 5-FU, whereas exosomal CA125 indicates metastatic disease [201]. Lin et al. indicated that EV-bound chemokine ligand 7 (CXCL7) could predict early response to chemotherapy in liver metastasis CRC since progressive disease showed increased EV-bound CXCL7 expression after chemotherapy [202].

Proteomic analysis of the serum-derived EVs unveiled secreted protein acidic and cysteine rich (SPARC) and leucine rich alpha-2-glycoprotein 1 (LRG1) can serve as diagnostic markers for colon cancer and predict tumor recurrence. Interestingly, EVs derived from tumors located in the right side of the colon demonstrate heightened expression of SPARC and LRG1 compared to EVs derived from tumors located in the left side of the colon [203]. Additionally, exosomal levels of beta-2-glycoprotein 1 (β2-GP1) and fibrinogen beta chain (FGB) are markedly higher in CRC than in paracancerous tissues and showed higher efficacy for early diagnosis compared to CEA and CA-19-9 [204]. Ganiga et al. reported that CAF-derived EVs display abundant EGF-like repeats and discoidin domains 3 (EDIL3), while NF-derived EVs exert suppressed quiescin sulfhydryl oxidase 1 (QSOX1) expression, and both markers are suppressed in CRC as opposed to corresponding controls [205] (Table 3).

**Table 3:** EV cargoes as diagnostic and prognostic biomarkers

EV cargo	Source/used sample	Expression patterns	Clinical implication	Refs.
***Non-coding RNAs***				
miR-21, let-7a, miR-1246miR-1229, miR-150, miR-223, and miR-23a	Serum; culture medium of CRC cell lines	Increased	Early diagnosis	[179]
miR-27a and miR-130a	Plasma	Increased	Diagnostic and Prognostic	[180]
miR-125a-3p and miR-320c	Plasma	Increased	Early diagnosis	[181]
miR-122	Serum and culture media	Increased	Diagnostic and prognostic	[182]
miR-193a and let-7 g	Plasma and culture media	Lower miR-193a and higher let-7 g expression in peritoneal metastasis	Diagnostic and prognostic	[183]
miR-23a and miR-301a	Serum	Increased	Early diagnosis	[184]
miR-99b-5p and miR-150-5p	Serum	Decreased	Diagnostic	[185]
miR-193a-5p	Plasma and culture media	Decreased	Diagnostic and prognostic	[187]
miR‐548c‐5p	Serum	Decreased	Diagnostic and prognostic	[192]
miR-6803-5p	Serum	Increased	Diagnostic and prognostic	[193]
miR-221	Plasma	Decreased	Prognostic	[194]
LNCV6_116109, LNCV6_98390, LNCV_108266, LNCV6_38772, LNCV6_84003, and LNCV6_98602	Plasma	Increased	Early diagnosis	[190]
LINC02418	Serum	Increased	Diagnostic	[191]
LncRNA GAS5	Plasma	Increased	Prognostic	[194]
lncRNA 91H	Plasma	Increased	Prognostic	[195]
lncRNA HOTTIP	Serum	Decreased	Prognostic	[196]
circLPAR1	Plasma and culture media	Decreased	Diagnostic and Prognostic	[186]
circ-PNN	Serum	Increased	Early diagnosis	[188]
hsa-circ-0004771	Serum	Increased	Early diagnosis	[189]
***Proteins***				
S100A9	Plasma	Increased	Diagnostic	[120]
Glypican-1 (GPC1)	Plasma and tumor tissues	Increased	Diagnostic	[199]
Copine III (CPNE3)	Plasma and tissue samples	Increased	Diagnostic and prognostic	[200]
Cytokeratin 19 (CK19), tumor-associated glycoprotein 72 (TAG72), and CA125	Plasma, tumor tissues, and cell culture supernatants	Increased	Diagnostic	[201]
EV bound chemokine ligand 7 (CXCL7)	Serum	Increased	Prognostic	[202]
SPARC and LRG1	Serum	Increased	Diagnostic and prognostic	[203]
Beta-2-glycoprotein 1 (β2-GP1) and fibrinogen beta chain (FGB)	Plasma and tissue samples	Increased	Early diagnosis	[204]
EDIL3 and QSOX1	Plasma, tissue samples, and culture media	Decreased	Early diagnosis	[205]

## sEVs promote CRC resistance against conventional and targeted therapy

Despite the diversification of therapeutic options for CRC patients, including surgery, chemotherapy, radiotherapy, targeted therapy, and immunotherapy, the development of acquired or intrinsic therapy resistance has been a crucial hurdle in successfully treating CRC.

Chemotherapy in the form of post-operative adjuvant therapy is widely acknowledged as the established approach for treating stage II and III CRC patients with an increased risk. 5-Fu and oxaliplatin are extensively utilized as the primary agents in first-line therapy for CRC [206]. Literature has elucidated EVs play essential roles in CRC resistance apart from conventional drug resistance [8]. For instance, exosomal miR-208b can attenuate CRC cells chemosensitivity to oxaliplatin through induction of Treg expansion by abrogating expression of PDCD4 in CD4 + T cells [207]. Abundantly expressed miR-196b-5p causes CRC cells stemness, thus contributing to 5-FU chemotherapy resistance through downregulating SOCS1 and SOCS3 and ultimately activating STAT3 signaling [208]. CRC-derived exosomal miR-210 are associated with anoikis resistance, EMT markers, and chemoresistance to 5-FU and treatment regimens similar to FOLFOX in cells with metastatic properties [209].

Exosomal secretion of Wnt by CAFs mediates the reprogramming of differentiated CRC cells via paracrine manner to promote CRC cells acquisition of stem-like properties and enhanced drug resistance [210]. Similarly, CAF-derived EVs transferring miR-92a-3p provoke CRC stemness and therapy resistance to 5-FU/oxaliplatin by negatively regulating FBXW7 and MOAP1 and eventually triggering Wnt/β-catenin pathway [34].

CAF-derived exosomes have been implicated in triggering a protective effect on CRC cells against oxaliplatin by activating the ERK/AKT pathway [211]. The transmission of lncRNA H19 through EVs between CAFs and CRC cells results in the acquisition of CSC phenotype and resistance to oxaliplatin through the stimulation of the Wnt/β-catenin. Mechanistically, lncRNA H19 acts as a ceRNA and sequestrates miR-141, thereby triggering Wnt/β-catenin signaling [212]. Additionally, Zhan et al. represented CAF-derived EVs containing circN4BP2L2 participate in CRC cell stemness and oxaliplatin resistance by escalating eukaryotic translation initiation factor 4A3 (EIF4A3) and triggering PI3K/AKT/mTOR pathway [213].

Chen and colleagues reported mitomycin-resistant CRC cells exert an augmented level of lncRNA HOTTIP, which is capable of being delivered to sensitive cells, thereby dampening their susceptibility to mitomycin. Indeed, HOTTIP exerts this effect by sponging miR-214 and upregulating karyopherin subunit alpha 3 (KPNA3) expression [214]. A study by Wang et al. unraveled that DNAJB8, an HSP40 family protein, is implicated in oxaliplatin resistance in colon cancer by stabilizing TP53 and diminishing its degradation and consequently upregulating MDR1. They also portrayed intercellular transmission of DNAJB8 via EVs and its potential in predicting colon cancer response to oxaliplatin [215]. By reprogramming glycometabolism of recipient cells, exosomal Isocitrate dehydrogenase 1 (IDH1) originated from 5-FU resistant CRC cells results in accelerated NADPH levels in sensitive cells, promoting their viability under 5-FU exposure [216].

Regarding targeted therapy with monoclonal antibodies, a study revealed the crucial involvement of UCA1-containing EVs in conferring resistance to cetuximab in CRC. These EVs are capable of being shuttled from cetuximab-resistant CRC cells to sensitive recipients, thus promoting the acquisition of reduced sensitivity to cetuximab [217]. Another study demonstrated EVs originated from cetuximab-resistant RKO colon cancer cell line potentiate Caco-2 cells escape from cetuximab toxicity by suppressing the PI3K negative regulator PTEN and upregulating Akt phosphorylation [218]. More recently, Yuan and colleagues elucidated the mechanism by which UCA1 contributes to the reduced efficacy of cetuximab treatment. They showed UCA1 counteracts the inhibitory effects of cetuximab on CRC cells by acting as a sponge for miR-495 and augmenting miR-495 downstream targets HGF and c-MET expression [219]. Tetraspanin 6 (Tspan6) deletion in Apc^min/+^ mice accelerates adenoma formation through autocrine activation of EGFR mediated by EV-associated secretion of the transmembrane form of TGF-α. Consistently, Tspan6-positive CRC patients manifest better responses to cetuximab [220].

Radiotherapy as a preoperative treatment approach is highly effective in reducing tumor load. Nevertheless, CRC patients often develop resistance to radiotherapy [221]. CAF secretion of exosomal miR-93-5p confers radiotherapy resistance in CRC by diminishing forkhead box A1 (FOXA1) and augmenting TGFB3 expression [222]. Additionally, CAF-derived EVs harboring miR-590-3p attenuate CRC cells sensitivity to radiotherapy through CLCA4 downregulation and subsequent PI3K/Akt signaling activation [221]. Moreover, CRC-derived exosomes exhibit significant upregulation of miR-19b, which imparts stem-like properties and radioresistance by exhausting FBXW7 expression and escalating Wnt/β-catenin signaling [223]. Another study unveiled circ_IFT80 is prominently elevated in EVs derived from the serum of CRC subjects and negatively correlates with radiosensitivity by sponging miR-296-5p and upregulating miR-296-5p target gene *MSI1* [224] (Table 4).

**Table 4:** Regulatory mechanism of EV cargoes in the development of therapy resistance in CRC

EV cargo	Producing cells	Expression patterns	Target cells	Related genes and/or pathways	Function/outcome	References
***Non-coding RNAs***						
miR-208b	Oxaliplatin-resistant colon cancer cells and mouse colon cancer cells; serum sample of CRC patients treated with FOLFOX	Increased	CD4 + T cells	Enhances Treg expansion via Inhibition of PDCD4 expression	Facilitates oxaliplatin resistance and tumor growth in vivo	[207]
miR-196b-5p	CRC cell lines; tissues and serum samples from CRC patients	Increased	CRC cells	Abolishes SOCS1 and SOCS3 expression and induces STAT3 signaling	Increased stemness and 5-FU resistance	[208]
miR-210	Colon cancer cells	Increased	Metastatic cells	NA	Contributes to anoikis resistance, EMT, and chemoresistance to 5-FU and FOLFOX-like treatment	[209]
miR-92a-3p	CAFs	Increased	LOVO CRC cells	Activates Wnt/β-catenin signaling and abolishes mitochondrial apoptosis by suppressing FBXW7 and MOAP1	Promotes stemness, EMT, and liver metastasis; 5-FU/oxaliplatin resistance	[34]
miR-21	CAFs	Increased	CRC cell lines	Activation of the ERK/AKT pathway	Oxaliplatin resistance and liver metastasis	[211]
miR-93-5p	CAFs	Increased	CRC cells	Targets FOXA1 expression and augments TGFB3	Reduced sensitivity to radiation and increased tumor growth in irradiated nude mice	[222]
miR-590-3p	CAFs	Increased	CRC cells	Inhibition of CLCA4 expression and increased PI3K/Akt signaling	Radiotherapy resistance and increased tumor growth	[221]
miR-19b	CRC cell line	Increased	CRC cells	Diminishes FBXW7 expression and stimulates Wnt/β-catenin signaling	Increased stemness and radioresistance	[223]
lncRNA H19	CAFs	Increased	CRC cells	ceRNA sponge for miR-141 and induces Wnt/β-catenin pathway	Promotes stemness and oxaliplatin resistance	[212]
lncRNA HOTTIP	Mitomycin-resistant CRC cells	Increased	CRC cells	Sponges miR-214 to elevate Karyopherin subunit alpha 3 (KPNA3) expression	Confers mitomycin resistance in sensitive cells	[214]
LncRNA-UCA1	Cetuximab-resistant colon cancer cells	Increased	Sensitive colon cancer cells	Suppresses PTEN expression and escalates Akt phosphorylation	Induction of cetuximab resistance in sensitive cells	[218]
LncRNA-UCA1	Cetuximab-resistant colon cancer cells	Increased	Cetuximab-sensitive colon cancer cells	Binds and inhibits miR-495 to augment hepatocyte growth factor (HGF) and c-MET expression, resulting in activation of AKT and MAPK pathways	Contributes to cetuximab resistance in vitro and in vivo	[219]
cricN4BP2L2	CAFs	Increased	CRC cells	Increased EIF4A3 expression and activation of PI3K/AKT/mTOR axis	Increased stemness and growth; inhibits apoptosis and potentiates oxaliplatin resistance	[213]
circ_IFT80	CRC cells; serum of CRC patients	Increased	CRC cells	Sponges miR-296-5p to elevate MSI1 expression	Increased proliferation and decreased apoptosis; Reduced radiosensitivity	[224]
***Proteins***						
DNAJB8	Oxaliplatin-resistant colon cancer cells; tissue and blood samples from colon cancer patients	Increased	Sensitive colon cancer cells	Interacts with TP53 to stabilize its level and consequently upregulates MDR1	Induction of oxaliplatin resistance in vitro and in vivo	[215]
Isocitrate dehydrogenase 1 (IDH1)	5-FU-resistant CRC cells	Increased	Sensitive CRC cells	Accelerates the glucose metabolism and escalates intracellular NADPH levels	Increased proliferation and 5-FU resistance	[216]
Transmembrane form of TGF-α (tmTGF-α)	APC^min/+^/Tspan6^−/−^ organoids	Increased	NA	Tetraspanin 6 (Tspan6) deletion in Apc^min/+^ mice results in autocrine activation of EGF-dependent signaling through secretion of EV-associated tmTGF-α	Reduced response to EGFR-targeted (cetuximab) therapy	[220]

## The effect of microbiota on CRC carcinogenesis

The gut microbiota refers to the set of microorganisms, such as bacteria, fungi, archaea, and viruses, predominantly composed of bacteria. Literature has established that the gut microbiota is defined as our hidden metabolic “organ” since it is involved in vast essential processes such as metabolism, digestion, development of the immune system, and bone homeostasis [225, 226]. As gastrointestinal cancers advance, there is a noticeable alteration in the composition of gut microorganisms, marked by an escalation in pathogenic bacteria and a decline in beneficial bacteria. Gut microbiota sustains homeostasis and immune function of the host, known as eubiosis, via secreted microbial metabolites or microbial EVs. On the other hand, dysbiosis of the gut microbiome hinders host homeostasis and inflammatory pathways, contributing to carcinogenesis [226].

### The effect of microbiota-derived sEVs on CRC carcinogenesis

Microbiota-derived EVs are crucial components in the intricate interplay between the gut microbiome and CRC carcinogenesis [227–229]. Yoon et al. used urine EVs of CRC patients and indicated gut microbiome displays a distinct composition compared with healthy corresponding, which is reflected in microbiota-derived EVs isolated from urine and shows potential for CRC diagnosis [229]. A study portrayed outer membrane vesicles (OMVs) derived from *Fusobacterium nucleatum subspecies polymorphum* activate Toll-like receptor 4 (TLR4) and affect the NF-κB pathway, thereby promoting intestinal inflammation by escalating the production of proinflammatory cytokines. HT-29 colon cancer cell line transfected with purified OMVs from *Fusobacterium nucleatum* manifests elevated IL-8 and TNF-α production [230]. *Bacteroides spp.* are regarded as one of the most consistently found groups of bacteria associated with CRC tumorigenesis, especially *Bacteroides fragilis*, which has been implicated in increased inflammatory response [231]. Kim et al. reported significant changes in the bacterial phyla *Firmicutes and Proteobacteria* in individuals with CRC, and microbiota-derived EVs harbor a diverse array of metabolic signals that reflects the host’s metabolism, nutritional condition, and immune function [227]. The outer membrane protein Fap2 derived from *Fusobacterium nucleatum* directly binds to the inhibitory receptor T cell immunoreceptor with immunoglobulin and ITIM domain (TIGIT) on NK cells and tumor-infiltrating T cells and abolishes their activity, thus enhancing tumor-immune evasion [232]. Ashrafian and colleagues demonstrated *Akkermansia muciniphila*-derived EVs attenuate inflammation by reducing TLR2 and TLR4 expression and enhancing the strength and function of the intestinal barrier by escalating the expression of tight junction proteins. Hence, *A. muciniphila*-derived EVs show potential as a viable therapeutic target for IBD [233]. *Fusobacterium nucleatum*-infected CRC cells-derived EVs harboring miR-1246/92b-3p/27a-3p induce migration ability in non-infected CRC cells by repressing glycogen synthase kinase 3 beta (GSK3β) expression. Concomitantly, CXCL16/RhoA/IL-8 are highly enriched in *F. nucleatum*-infected CRC cells and provoke migration ability and metastasis via CXCL16/CXCR6 axis [228]. *Pediococcus pentosaceus*-derived EVs modulate host immunity and exhibit anti-inflammatory effects by inducing M2-like macrophage polarization in macrophages and MDSCs differentiation in a TLR2-dependent manner. Dextran sodium sulfate (DSS)-induced colitis model abolished colon shortening and disruption of crypt architecture after treatment with *P. pentosaceus*-secreted EVs [234].

### The effect of microbiota on autophagy in CRC carcinogenesis

Emerging studies link dysbiosis of the gut microbiota to dysregulation of autophagy and altered immune responses in the development and metastasis of CRC [235–237]. Inhibition of ATG7 dampens intestinal tumorigenesis in *Apc*^+*/−*^ mice by stimulating adaptive immunity and promoting efficient infiltration of CD8 + T cells. The depletion of ATG7 causes intestinal dysbiosis and such alterations in gut microbiota in Atg7-deficient mice are necessary for CD8 + T cell anticancer response [235]. *F. nucleatum* infection has been implicated in the metastasis of CRC through the upregulation of caspase activation and recruitment domain 3 (CARD3) and autophagy flux. *F. nucleatum* elevates LC3-II expression, autophagosome biogenesis, and induces the expression of beclin1, ATG5, and ATG7 in CRC cells [236]. Additionally, *F. nucleatum* is enriched in subjects with recurrent CRC and is linked to chemotherapy resistance. This effect is attributed to F. nucleatum’s capability to suppress TLR4 and MYD88 and downregulate miR-18a* and miR-4802, resulting in autophagy activation [237]. Another study demonstrated that colon cancer cells infected with cytotoxin-associated gene A (CagA)-positive *H. pylori* depicted escalated autophagy flux, growth, and invasion. CagA positive-*H. pylori* stimulates autophagy pathways by depleting miR-125b-5p and increasing LC3B-II/LC3B-I and beclin-1 expression in colon cancer cells [238].

## Therapeutic application of sEVs in CRC

Given the significant involvement of EVs in the initiation, metastasis, and development of tumor resistance, they present appealing targets for therapeutic interventions against CRC. Despite emerging progress in CRC treatment, such as immunotherapy and targeted therapy, chemoresistance and cancer recurrence still happen. Hence, efficient treatment of CRC patients, especially those in an advanced stage, requires exploring new therapeutic strategies [239].

Over the past decade, there have been endeavors to harness the potential of EVs for the treatment of CRC, resulting in the advancement of therapies based on EVs. Different approaches can be used to reduce EV-related adverse effects in CRC. Some researchers have postulated inhibiting EV biogenesis and release can abrogate CRC progression and therapy resistance. Depletion of TSG101, a component of the ESCRT complex, suppressed Wnt5b-associated EV release and abolished Wnt5b-dependent cell growth in Caco-2 cells [240]. Inhibition of EVs-associated miR-19b secretion by GW4869, an inhibitor of nSMase2, suppresses oxaliplatin resistance in CRC [241]. Similarly, EVs derived from CRC cells abundantly expressing calcium-dependent activator protein for secretion 1 (CAPS1) stimulate the migratory capacity of normal colonic epithelial cells, and inhibition of EV secretion by GW4869 abolishes this effect [242]. However, inhibition of nSMase2 leads to only a partial reduction of EVs. As stated before, Rab-GTPase families are involved in EV release; thus, inhibiting Rab proteins can block EV secretion [243]. Rab27A knockdown has been illustrated to curtail growth and invasion of CRC cells, while Rab27A overexpression restored this effect [244]. Sulfisoxazole, a sulfonamide antibacterial, disrupts EVs biogenesis and attenuates exosomal PD-L1 expression by reducing Rab27A in colon cancer cells, thereby synergistically improving anti-PD1 therapy response [245].

Other groups proposed altering EVs uptake as another strategy for targeting EVs in cancer. Methyl-β-cyclodextrin (MβCD), a Caveolin-mediated endocytosis inhibitor, exhausts cholesterol in the plasma membrane and hinders lipid rafts, ultimately inhibiting EVs uptake [246]. MβCD interferes with EVs uptake by glioblastoma cells [247]. Additionally, Tu et al. indicated silencing of proteins involved in the process of endocytosis, such as clathrin heavy chain, caveolin-1, flotillin-1, and dynamin-2, attenuates the uptake of EVs originating from bone marrow-derived stromal cell (BMSC) in multiple myeloma cells and abolishes EVs-induced bortezomib resistance [248].

Recently, studies have been mainly focused on utilizing EVs as natural vehicles to effectively transfer therapeutic agents into TME. EVs are natural biological carriers that can efficiently transfer drugs to cancer cells with less immunogenicity and toxicity than other delivery systems, such as metal, liposome, and polymer nanoparticles, and they can specifically target cells with specific proteins. Thanks to their endogenous lipid membrane composition, EVs can efficiently distribute in tissues and cross anatomical barriers [249]. Scientists have developed bioengineered EVs with the purpose of delivering anti-neoplastic drugs and functional RNA molecules, such as miRNA and siRNA, to selectively target neoplastic cells. Li et al. demonstrated EVs loaded with doxorubicin display better efficacy in inhibiting CRC growth in tumor-bearing mice than free doxorubicin, extending mice survival with reduced cardiotoxicity [250]. Administration of EVs co-delivering miR-21 inhibitor oligonucleotide and 5-FU to 5-FU-resistant CRC cells in tumor-bearing mice depicted diminished chemoresistance and enhanced apoptosis by restoring PTEN and human MutS homolog 2 (hMSH2) expression [251]. A study highlighted that EV-associated miR-25-3p triggers PMN formation by provoking vascular leakage and tubulogenesis, suggesting silencing miR-25-3p can circumvent this effect [48]. miR-214 is notably downregulated in radioresistant CRC specimens and exerts a repressive effect on autophagy via its downstream target ATG12, indicating restoring miR-214 expression can enhance CRC radiosensitivity by exhausting radiation-induced autophagy [252]. Exosomal Wnt derived from fibroblast confer differentiated CRC cells stem-like characteristics resulting in chemoresistance, thus inhibiting exosomal Wnt reversed this effect in vitro and in vivo [210].

EVs have been recruited as a cell-free vaccine to enhance the immune response against tumors and restrict the growth of malignant cells. In a pioneering study conducted by Dai and colleagues, a clinical trial in the initial phase depicted immunization with EVs isolated from ascites of CRC subjects followed by immunotherapy with granulocyte–macrophage colony-stimulating factor (GM-CSF) induced a safe and well-tolerated response from tumor-specific cytotoxic T cells in CRC patients [253]. Phase I trials portrayed the safety and feasibility of dendritic cells-derived EVs in melanoma and non-small cell lung cancer (NSCLC) patients and their capability to enhance NK cell cytolytic functions [254, 255]. Additionally, the phase II trial depicted DC-derived EVs as a maintenance immunotherapy in chemotherapy-responding NSCLC patients to boost NK cells function in an NKp30-dependent manner. Moreover, enhanced NK cells function positively correlates with MHC-II and BAG6 expression in the final vaccine product [256]. More recently, Meng et al. demonstrated immunization with embryonic stem cells (ESCs)-derived EVs expressing GM-CSF prevents lung metastasis effectively in Lewis lung carcinoma-challenged mice compared to non-vaccinated tumor-bearing mice. Further, immunization with ESC-EV/GM-CSF circumvents tumor-infiltrating Tregs, MDSCs, and TAMs and provokes cytokine secretion from intratumor CD8 + T cells [257].

## Conclusion

Studies have shown that EVs are involved in different steps of CRC formation and metastasis via orchestrating bidirectional signaling between CRC cells, stromal cells, and immune cells in TME. EVs contribute to CRC invasion and metastasis by establishing a tumor-favorable niche in a secondary site, inducing EMT, triggering angiogenesis, and conducting an immune-suppressive environment. Hence, it is crucial to study the underlying processes through which EVs contribute to CRC metastasis formation to devise new treatment strategies. Although numerous studies identified a wide variety of EV cargoes for CRC diagnosis and prognosis prediction, EV heterogeneity and microscopic size, isolation, purification, characterization, and diversity of experimental models are major challenges in their translation in clinical practice. Autophagy and EV secretion are both implicated in CRC progression and are linked together since they share common pathways and molecular machinery. The context of secretory autophagy further supports a crucial interaction between these processes. However, the precise mechanism underlying their crosstalk in CRC progression requires further investigation.

## Data Availability

Not applicable.

## Abbreviations

EVs: Extracellular vesicles
CRC: Colorectal cancer
TME: Tumor microenvironment
EMT: Epithelial-mesenchymal transition
sEVs: Small EVs
ILVs: Intraluminal vesicles
MVB: Multivesicular body
ESCRT: Endosomal sorting complex required for transport
PM: Plasma membrane
SNAREs: Soluble N-ethylmaleimide-sensitive factor attachment proteins receptors
VAMP: Vesicle-associated membrane protein
HSP70: Heat shock protein 70
TSG 101: Tumor susceptibility 101
AF4: Asymmetric flow field-flow fractionation
VPS: Vacuolar protein-associated protein
AP: Adaptor-related protein
ECM: Extracellular matrix
MET: Mesenchymal-epithelial transition
ZEB: Zinc-finger E-box binding
FZD: Frizzled protein
TUBB3: β-III tubulin
PDCD4: Programmed cell death 4
CAFs: Cancer-associated fibroblasts
SOCS1: Suppressor of cytokine signaling 1
ZBTB2: Zinc finger and BTB domain containing 2
CXCR: CXC Chemokine Receptor
CXCL: CXC Chemokine Ligand
L-OHP: Oxaliplatin
ANXA2: Annexin A2
PMN: Premetastatic niche
BMDCs: Bone marrow-derived cells
TAMs: Tumor-associated macrophages
Tregs: Regulatory T cells
TANs: Tumor-associated neutrophils
KLF2: Krüppel-like factor 2
KLF4: Krüppel-like factor 4
VEGFR2: Vascular endothelial growth factor receptor 2
IRF2: Interferon regulatory factor 2
VEGFC: Vascular endothelial growth factor C
CTC: Circulating tumor cells
PDAC: Pancreatic ductal adenocarcinoma
HSCs: Hepatic stellate cells
TGF- β: Transforming growth factor-β
TGFBR3: TGF-beta receptor III
MIF: Migration inhibitory factor
MMP: Matrix metalloproteinase
NK: Natural Killer
TNF-α: Tumor necrosis factor alpha
IFN-γ: Interferon gamma
ITGBL1: Integrin beta-like 1
TLR7: Toll-like receptor 7
IL-6: Interleukin 6
ACLY: ATP-citrate lyase
HIF1α: Hypoxia-inducible factor-1α
HUVECs: Human umbilical vein endothelial cells
KRIT1: Krev interaction trapped protein 1
Ccnd1: Cyclin D1
AA-TKIs: Antiangiogenic tyrosine kinase inhibitors
EPCs: Endothelial progenitor cells
APC: Adenomatous polyposis coli
MAPK: Mitogen-activated protein kinase
PIGF: Placenta growth factor
EGF: Epidermal growth factor
DUSP2: Dual specificity protein phosphatase 2
ANGPTL1: Angiopoietin-like protein 1
SDF 1: Stromal cell derived factor 1
HGF: Hepatocyte growth factor
FAP: Fibroblast activation protein
RECK: Reversion-inducing cysteine-rich protein with kazal motifs
BAP31: B-cell receptor-associated protein 31
Smad2: Mothers against decapentaplegic homolog 2
Smad3: Mothers against decapentaplegic homolog 3
MVP: Major vault protein
CCND2: Cyclin D2
PLAGL2: Pleomorphic adenoma gene-like 2
IGF-2: Insulin-like growth factor-2
TRAF3: TNF receptor-associated factor 3
hTERT: Human telomerase reverse transcriptase
DCs: Dendritic cells
ROS: Reactive oxygen species
ZC3H12B: Zinc-finger-type-containing 12B
MDSCs: Myeloid-derived suppressor cells
PEDF: Pigment epithelium-derived factor
CMA: Chaperone-mediated autophagy
BRCA1: Breast cancer 1
ATGs: Autophagy-related genes
BECN1: Beclin-1
UVRAG: Ultraviolet irradiation resistance-associated gene
BIF-1: Bax-interacting factor-1
PI3K: Phosphoinositide 3-kinase
mTOR: Mammalian target of rapamycin
PTEN: Phosphatase and tensin homolog
AMPK: AMP-activated protein kinase
DRAM1: DNA damage-regulated autophagy modulator 1
CSCs: Cancer stem cells
HCC: Hepatocellular carcinoma
5-Fu: 5-Fluorouracil
SA: Secretory autophagy
LC3β: Light chain 3beta
α-SMA: Alpha-smooth muscle actin
ALIX: Alg-2 interacting protein-X
CML: Chronic myeloid leukemia
RBPs: RNA-binding proteins
mTORC1: Mechanistic target of rapamycin complex 1
sno-RNAs: Small nucleolar RNAs
ceRNA: Competing endogenous RNA
miRNAs: MicroRNA
ncRNAs: Non-coding RNAs
lncRNA: Long non-coding RNA
siRNA: Small interfering RNA
circRNA: Circular RNA
LDELS: LC3B-dependent EV loading and secretion
nSMase2: Neutral sphingomyelinase 2
FAN: Factor-associated with nSMase2
TMED10: Transmembrane p24-trafficking protein 10
CEA: Carcinoembryonic antigen
BRD4: Bromodomain-containing protein 4
eIF3h: Eukaryotic translation initiation factor 3 subunit h
METTL3: Methyltransferase-like 3
CUX1: CUT-like homeobox 1
ITSN1: Intersectin 1
HNRNPK: Heterogeneous nuclear ribonucleoprotein K
PC: Phosphatidylcholine
PE: Phosphatidylethanolamine
PI: Phosphatidylinositol
SM: Sphingomyelin
HexCer: Hexosylceramide
GPC1: Glypican-1
CPNE3: Copine III
CK19: Cytokeratin 19
TAG72: Tumor-associated glycoprotein 72
SPARC: Secreted protein acidic and cysteine rich
LRG1: Leucine rich alpha-2-glycoprotein 1
β2-GP1: Beta-2-glycoprotein 1
FGB: Fibrinogen beta chain
EDIL3: EGF-like repeats and discoidin domains 3
QSOX1: Quiescin sulfhydryl oxidase 1
FBXW7: F-box and WD repeat domain containing 7
MOAP1: Modulator of apoptosis 1
EIF4A3: Eukaryotic translation initiation factor 4A3
KPNA3: Karyopherin subunit alpha 3
IDH1: Isocitrate dehydrogenase 1
UCA1: Urothelial carcinoma-associated 1
Tspan6: Tetraspanin 6
FOXA1: Forkhead box A1
OMVs: Outer membrane vesicles
TIGIT: T cell immunoreceptor with immunoglobulin and ITIM domain
GSK3β: Glycogen synthase kinase 3 beta
DSS: Dextran sodium sulfate
CAPS1: Calcium-dependent activator protein for secretion 1
MβCD: Methyl-β-cyclodextrin
BMSC: Bone mesenchymal stem cell
hMSH2: Human MutS homolog 2
GM-CSF: Granulocyte–macrophage colony-stimulating factor
NSCLC: Non-small cell lung cancer
ESCs: Embryonic stem cells
SAPK: Stress-activated protein kinase
TRAIL: Tumor necrosis factor-related apoptosis inducing ligand
